# Expression of Annexin A2 Promotes Cancer Progression in Estrogen Receptor Negative Breast Cancers

**DOI:** 10.3390/cells9071582

**Published:** 2020-06-30

**Authors:** Amira F. Mahdi, Beatrice Malacrida, Joanne Nolan, Mary E. McCumiskey, Anne B. Merrigan, Ashish Lal, Shona Tormey, Aoife J. Lowery, Kieran McGourty, Patrick A. Kiely

**Affiliations:** 1 Graduate Entry Medical School, University of Limerick, V94 T9PX Limerick, Ireland; amira.mahdi@ul.ie (A.F.M.); b.malacrida@qmul.ac.uk (B.M.); joanne.nolan@ul.ie (J.N.); 2 Health Research Institute, University of Limerick and Bernal Institute, University of Limerick, V94 T9PX Limerick, Ireland; kieran.mcgourty@ul.ie; 3 Stokes Laboratories, Bernal Institute, University of Limerick, V94 T9PX Limerick, Ireland; mary.mccumiskey@ul.ie; 4 Department of Surgery, University Hospital Limerick, V94 F858 Limerick, Ireland; anne.merrigan@hse.ie (A.B.M.); ashish.lal@hse.ie (A.L.); shona.tormey@hse.ie (S.T.); 5 Lambe Institute for Translational Research, National University of Ireland Galway, H91 TK33 Galway, Ireland; aoife.lowery@nuigalway.ie; 6 Department of Chemical Sciences, University of Limerick, V94 T9PX Limerick, Ireland

**Keywords:** breast cancer, estrogen receptor negative, Annexin A2, mass spectrometry, metastasis

## Abstract

When breast cancer progresses to a metastatic stage, survival rates decline rapidly and it is considered incurable. Thus, deciphering the critical mechanisms of metastasis is of vital importance to develop new treatment options. We hypothesize that studying the proteins that are newly synthesized during the metastatic processes of migration and invasion will greatly enhance our understanding of breast cancer progression. We conducted a mass spectrometry screen following bioorthogonal noncanonical amino acid tagging to elucidate changes in the nascent proteome that occur during epidermal growth factor stimulation in migrating and invading cells. Annexin A2 was identified in this screen and subsequent examination of breast cancer cell lines revealed that Annexin A2 is specifically upregulated in estrogen receptor negative (ER-) cell lines. Furthermore, siRNA knockdown showed that Annexin A2 expression promotes the proliferation, wound healing and directional migration of breast cancer cells. In patients, Annexin A2 expression is increased in ER- breast cancer subtypes. Additionally, high Annexin A2 expression confers a higher probability of distant metastasis specifically for ER- patients. This work establishes a pivotal role of Annexin A2 in breast cancer progression and identifies Annexin A2 as a potential therapeutic target for the more aggressive and harder to treat ER- subtype.

## 1. Introduction

All cells, including tumor cells, exist not in a vacuum but in a continuous, interconnected and unbounded network of relationships. This includes relationships with other cells and with the extracellular or stromal environment in which the cells reside. In the case of tumor cells, these surroundings are collectively known as the tumor microenvironment (TME) [1]. Understanding how the TME can affect cellular behavior is of particular interest in attempts to elucidate the cellular mechanisms that enable a metastatic phenotype. This is because two of the cellular behaviors essential to the process of metastasis (migration and invasion) rely on interactions between the tumor cell and its surrounding TME [2,3]. In migration, cells gain the ability to become aberrantly motile. This motility is given directionality via chemotaxis in which a receptor expressed by the cancer cells interacts with a chemoattractant ligand present within the TME [4]. Invasion, referred to as the defining feature of malignancy, is the ability of cancer cells to change their morphology and alter their surroundings in order to penetrate the basement membrane, degrade encompassing stroma and escape usual tissue boundaries [5]. These pivotal mechanisms of cancer disease progression can be modelled in vitro using cell lines and well-defined transwell migration and invasion assays [6].

Deciphering the steps involved in the metastatic process is of vital importance in breast cancer. Breast cancer begins as a disease localized to the breast tissue. Advancements in multi-modal treatments such as surgery and radiation, have increased the chances for cure for over 70% of patients [7]. However, the mortality associated with the disease of breast cancer does not arise from these localized breast tumors but rather, from when the cancer undergoes metastasis and spreads to distant sites and colonizes vital organs of the body. At present, metastatic breast cancer is considered incurable. Once this stage of disease is reached, treatment aims to prolong survival and control symptoms. For this reason, there is an urgent need to decipher the specific environmental cues and resultant mechanisms involved in the metastatic process. This is of particular importance in estrogen receptor (ER) negative breast cancer subtypes (including both the triple negative (TNBC) and HER2+ groupings) as these tumors have a more aggressive phenotype and higher rate of distant metastasis than ER positive tumors [8]. This understanding will guide the development of interventions aimed at preventing the metastasis and associated mortality seen in those diagnosed with ER negative breast cancer.

One method to assess the effect of environmental cues is to study the resulting influence on the cell’s proteome [9]. There is strong evidence that proteomic analysis offers a truer physiological insight over transcriptomic methods. Measurements of mRNA transcript abundance alone have been reported to be a poor predictor of protein synthesis due to the fact it is subject to an independent layer of translational control that controls protein expression [10]. Transcriptomics also fails to take into account protein turnover and degradation as well as activation state through post-translational modification. Proteomic analysis of breast cancers has revealed prognostic markers [11], novel subtype classifications [12] and targetable protein abundance differences as cancer progresses through clinical stages [13]. Analysis of proteins that are newly translated and synthesized by cells allows for an accurate snapshot of cellular activities during a specific process or duration. In fact, the execution of cancer hallmarks—such as migration and invasion—which drive progression are ultimately achieved by dynamic alterations in protein expression and post-translational modifications of those proteins [14].

With this in mind, we have developed a model to investigate changes in the nascent proteome of aggressive, ER negative breast cancer cells as they undergo migration and invasion, stimulated through modulation of the extra-cellular environment. In this study, we utilized these in vitro transwell models in combination with bioorthogonal noncanonical amino acid tagging (BONCAT) to investigate proteomic changes in MDA-MB-231 breast cancer cells as they undergo epidermal growth factor (EGF) mediated migration and invasion. BONCAT is a widely used method to assess the primary nascent proteomic response to a specific stimulus within a specific short time period [9]. This method allowed us to isolate and identify newly synthesized proteins that were translated in the two hours following directional EGF stimulation. Metastasis is a dynamic process of changes in cellular expression and behaviors and thus, must be investigated via a dynamic and temporal model. Isolating the nascent proteome in this manner facilitates the precise examination of the cancer cell’s translational response to environmental cues, which trigger a metastatic phenotype. This knowledge may elucidate novel therapeutic strategies or treatments that prevent the metastatic spread of cancer cells.

## 2. Materials and Methods

### 2.1. Cell Culture

Human breast cancer cell lines (MDA-MB-231, MCF-7, ZR-75-1) were purchased from the ECACC culture collection (Sigma Aldrich, Wicklow, Ireland). Cell lines were routinely tested upon freezing and thawing for mycoplasma contamination using PCR. Cells were maintained at 37 °C, 5% CO_2_ humidified incubator in DMEM-high glucose (MDA-MB-231 & MCF-7) or RPMI (ZR-75-1) supplemented with 10% fetal bovine serum, 5% l-glutamine and 5% penicillin/streptomycin (all obtained from Sigma Aldrich). ZR-75-1 media was additionally supplemented with 5% sodium pyruvate (Sigma Aldrich). For 3-dimensional culture, the on-top method was used where cells were seeded into wells containing Growth-Factor Reduced Matrigel™ (Biosciences, Dublin, Ireland) then covered with DMEM supplemented with 2% Matrigel. For analysis of cell signaling, cells were washed with PBS before being covered with serum free medium for 4 h prior to EGF stimulation (Peprotech, London, UK).

### 2.2. Click-iT Reaction

Cell cultures plated in 2-D (24 h) and 3-D (6 day) on transwell membranes were starved of serum and methionine for 4 h. After this, serum free, methionine free DMEM supplemented with 50 ng/mL EGF was placed into the lower chamber. At the same time, the upper chamber media was replaced with serum free, methionine free DMEM supplemented with 50 μM Azido Homo Alaine (AHA) (Thermo Fisher, Dublin, Ireland), a methionine analogue that is incorporated into newly synthesized proteins. Cells were then allowed to migrate and invade for 2 h. Cells were harvested from Matrigel or scraped from the membrane then lysed. The Click-iT reaction and incorporation of the tetramethylrhodamine (TAMRA) tag was then carried out according to Click-iT TAMRA Protein Analysis Detection Kit protocol (Thermo Fisher) to isolate newly synthesized proteins which had incorporated the AHA labelling. A control replicate omitting the EGF supplementation was carried out for both 2-D and 3-D cultures.

### 2.3. Immunoprecipitation

Newly synthesized proteins that had incorporated the TAMRA residue were then isolated from total cell lysate using anti-TAMRA immunoprecipitation. Lysates were precleared with protein G agarose beads (Roche, via Sigma Aldrich). The supernatant was then incubated with Anti-TAMRA antibody (Thermo Fisher) and protein G agarose beads at 4 °C overnight. The beads were washed with cell lysis buffer before being boiled with SDS loading buffer for 5 min. Immunoprecipitated samples were resolved on a 12% SDS gel and stained with Coomassie Instant Blue (Expedeon, Cambridge, UK). A control containing only beads was also performed to account for non-specific binding and contaminant exclusion.

### 2.4. Mass Spectrometry and Data Analysis

Following SDS-PAGE, gel lanes containing protein bands stained with Coomassie were excised from the gel and placed in 20% glycerol for transport to Mass Spectrometry and Proteomics Facility, University of St Andrews, Fife. Gel chunks were subjected to trypsin digestion. Next, proteolytic peptide mixtures were separated by nano-LC utilizing an Eksigent two-dimensional LC NanoLC system (Eksigent/Applied Biosystems Sciex, MA, USA) interfaced with a QStar XL mass spectrometer (Applied Biosystems Sciex, MA, USA). Data sets were searched against the NCBInr 20160830 database using MASCOT software (Matrix Science, MA, USA) under the following parameters: maximum one missed cleavage of trypsin digestion, carbamidomethyl (C) as a fixed modification, oxidation (M) as a variable modification, a peptide mass tolerance of ± 20 ppm and a fragment mass tolerance of ±0.05 Da. Only scores higher than the significance threshold (*p* < 0.05) were reported. Proteins identified in bead only and non-EGF supplemented controls were subtracted from the protein lists. Proteins were then characterized functionally based on the available literature associated with their NCBI entry. Gene ontology (Molecular Function–Slim) enrichment analysis was carried out using the Panther overrepresentation test (Version 14.1) [15] with the Fisher’s exact test to calculate *p*-value and Benjamini–Hochberg procedure to calculate false discovery rate (FDR). FDR cut-off was <0.05. Protein-protein interaction networks were probed using STRING v11 [16] and visualized using Cytoscape StringApp [17].

### 2.5. Cell Lysis, SDS PAGE and Western Blotting

The cells were lysed in 1% NP-40 lysis buffer then quantified and denatured with SDS loading buffer and boiled for 5 min. Lysates were separated on 12% SDS acrylamide gels and subsequently transferred to nitrocellulose membrane. Membranes were blocked using 5% BSA for 1 h at room temperature then probed with corresponding primary antibodies at 4 °C overnight. Dilutions of antibodies were as follows: Annexin A2 1:1000 (Abcam, Cambridge UK), Annexin A2 1:1000 (BD Bioscience), B-Actin 1:1000 (Sigma), phospo-Tyr24-Annexin A2 1:250 (Santa Cruz Biotechnology, Heidelberg, Germany) E-Cadherin 1:1000 (Abcam). IRdye700- or IRdye800-conjugated secondary antibodies, were then coupled to the primary antibody for 1 h at room temperature. Protein bands were detected using the Odyssey Sc (LI-COR, Cambridge, UK).and quantified using Image Studio 5.2 (LI-COR)).

### 2.6. siRNA Transfection

To reduce Annexin A2 expression, the Neon Transfection System (Thermo Fisher) was used to transiently transfect MDA-MB-231 cells with siRNA oligonucleotides targeting ANXA2 mRNA according to the protocol supplied by Neon. Two different siRNA oligonucleotides were obtained from Qiagen (Manchester, UK) and used at 10 nM for 72 h (Sequences reported in Table S3). MISSION^®^ siRNA Universal Negative Control (Sigma Aldrich) was transfected as negative control.

### 2.7. Tissue Collection

Ethical approval was granted by the University Hospital Limerick’s Ethics Committee and was allocated the ethical approval numbers 22/14 and 141/12. From the resected surgical specimens, core biopsies from the tumor mass were extracted by the operating surgeon. Where possible, pathologically normal breast tissue samples were also collected. The samples were stored in Allprotect Tissue Reagent (Qiagen) at −80 °C until extraction.

### 2.8. RNA Extraction

RNA from breast cancer cell lines was extracted using RNeasy Plus Mini kit (Qiagen) according to manufacturer’s instructions. For breast tissue samples, RNA was extracted by submerging tissue into liquid nitrogen, grinding into a fine powder then using the RNeasy Lipid Tissue Mini Kit (Qiagen) in combination with Maxtract tubes (Qiagen) according to manufacturer’s instructions. RNA was quantified using Nanodrop (Thermo Fisher) and quality was assessed by agarose gel separation. cDNA was then synthesized from 1 µg or 500 ng of total RNA using a Vilo cDNA kit (Invitrogen, via Biosciences, Dublin, Ireland).

### 2.9. RT-qPCR

Applied Biosystems QuantStudio 7 Flex Real-Time PCR was used to measure gene expression using Taqman Gene Expression Assay Kits (Thermo Fisher). Seven different housekeeping genes were tested using Normfinder and the three most stable genes between the three cell lines were used to normalise gene expression across samples as previously described [18]. Relative fold change in gene expression was calculated using the 2^−ΔΔCT^ method and represented relative to a control sample set to 1 for each experiment. Data presented is from three independent experiments displayed as mean +/− SEM.

### 2.10. Cell Migration Assays

For wound healing assays, transfected cells were seeded into cell culture wells containing ibidi inserts (ibidi GmbH, Gräfelfing, Germany). After 72 h incubation, the insert was carefully removed and cells imaged using Olympus cellSens Dimension 1.12. Cells were incubated for 24 h, after which images of the wound closure were taken. Six fields of view were imaged for each condition and percent wound closure was measured using Image J (version 1.52n). Three individual experiments were carried out to verify results. Data is displayed as mean across three individual experiments +/− SEM.

For directional migration assays, the underside of 8 µm transwell membranes were coated with fibronectin before 6 × 10^5^ cells in serum free DMEM media were plated onto the upper chamber. Cells were allowed to migrate towards a lower chamber containing 50 ng/mL EGF in serum free media for 24 h. Cells were removed from the upper chamber of the membrane. Migrated cells were fixed and stained with crystal violet and imaged. To quantify the cell migration, crystal violet stain was dissolved 10% acetic acid and intensity measured as absorbance at 595 nm in triplicate. Three individual experiments were carried out to verify results.

### 2.11. Real-Time Proliferation Assay

The rate of proliferation was monitored in real time using the xCELLigence RTCA DP E-plate system (ACEA, CA, USA). 1.5 × 10^4^ MDA-MB-231 cells transfected either with ANXA2 siRNA or negative control RNA were seeded into each well. The impedance value of each well was measured by the xCELLigence system every 15 min for 96 h and expressed as a cell index value (CI). CI was normalized at 4 h post seeding. Wells were analyzed in duplicate. Three individual experiments were carried out to verify results. Data is displayed as mean cell index +/− SEM.

### 2.12. Use of Publicly Available Gene & Protein Expression Datasets

Protein expression data of breast cancer cell lines was provided by D P Nusinow et al. [19]. Protein expression was measured using quantitative mass spectrometry and is expressed as normalized, relative values of protein abundance. Full details of normalization is available at [20]. Gene expression data of breast cancer cell lines was downloaded from the Cancer Cell Line Encyclopedia (CCLE) (Available at: https://portals.broadinstitute.org/ccle; GEO Series GSE36139). This data set was collected using GPL15308 Affymetrix Human Genome U133 Plus 2.0 Array. Microarray data was evaluated using the robust multichip average method (RMA) and quantile normalized before being made available through the CCLE [21]. Breast cancer cell lines were then classified by ER expression (positive or negative) according to the available literature [22,23] and AnxA2 mRNA expression was compared between groups. Dataset GSE42568 [24] was downloaded from the GEO data repository (http://www.ncbi.nlm.nih.gov/geo) using the GEOR function. This data set was collected using GPL570 [HG-U133_Plus_2] Affymetrix Human Genome U133 Plus 2.0 Array. Microarray data was evaluated using the GC robust multichip average (GCRMA) method and quantile normalized before being made available through GEO. Protein expression data using SILAC quantitative mass spectrometry of 40 breast cancer tissue samples was obtained from Tyanova et al. (2016) [25]. Normalized protein expression depicted as log2 (ratio of L/H). Distant metastasis free survival (interval from surgery to date of metastasis diagnosis) was investigated by downloading data from http://kmplot.com, a manually curated database which collates gene expression and survival information from GEO, EGA and TCGA datasets (35 breast cancer datasets, using Affymetrix HGU133A and HGU133 Plus 2.0 microarrays) [26]. The median ANXA2 expression value for each cohort was chosen as the high/low cut off point. Graphpad Prism 8 was then used to produce Kaplan–Meier graphs and to calculate log-rank test result and hazard ratios.

### 2.13. Statistical Analysis

Statistical analyses were performed using GraphPad Prism version 8.0.2. The Shapiro–Wilk test was used to assess normality of sample distribution. Differences between two groups were calculated using Student’s T-test (normally distributed samples) or Mann Whitney U test (non-normally distributed samples). Differences between three or more groups was calculated using ANOVA and multiple comparisons were performed using Bonferroni Correction. Data was reported as mean +/− standard deviation for technical replicates or mean +/− standard error of mean for biological replicates. A *p* value of <0.05 was considered statistically significant. Within figures, asterisks denote significance levels as such: * *p* ≤ 0.05; ** *p* ≤ 0.01; *** *p* ≤ 0.001.

## 3. Results

### 3.1. Isolation and Identification of Newly Synthesised Proteins Involved in Breast Cancer Cell Metastasis

**Figure 1:**
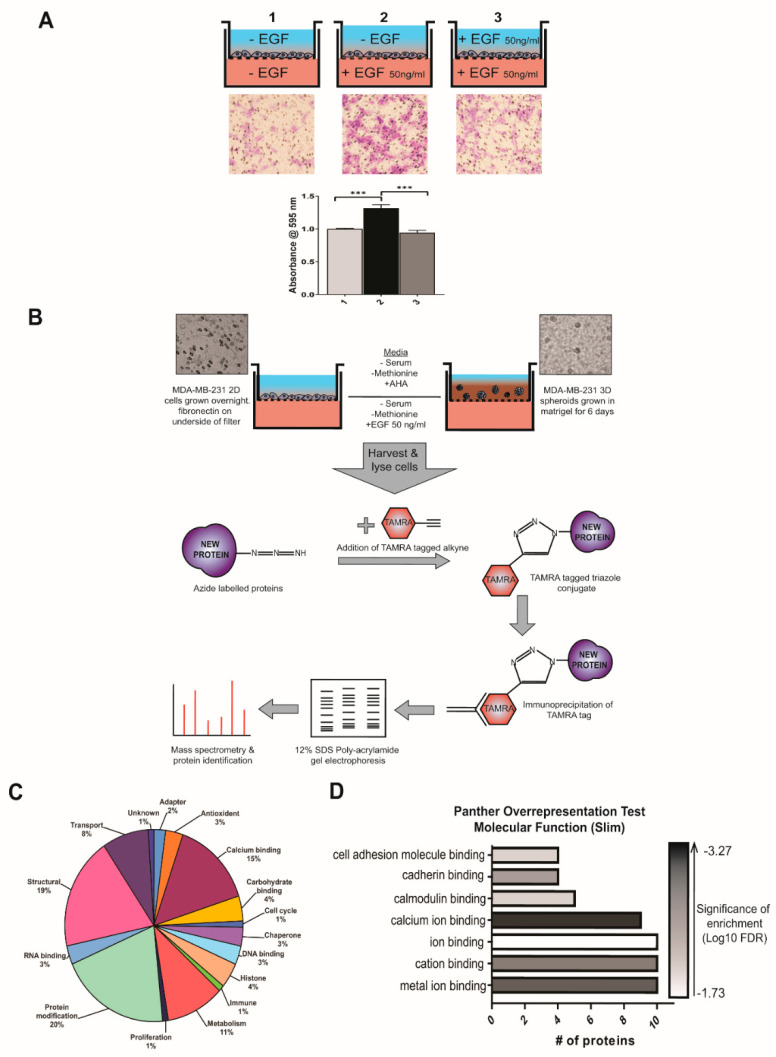
Isolation, identification, and functional characterization of newly synthesized proteins in MDA-MB-231 migration and invasion. (**A**) MDA-MB-231 cells were plated in the upper chamber of transwell plates and media was supplemented (+/− EGF) according to diagram. Cell migration after 4 h was measured by crystal violet (CV) staining of cells which moved through the well and adhered to the underside of the membrane. Non-migrated cells were removed prior to staining. Membranes were then imaged using inverted microscope and migration of cells was quantified by dissolving of CV stain and measuring absorbance at 595 nm. Data displayed as mean ± SEM, of 3 independent experiments, CV absorbance normalized to average value for well 1. Statistical analysis by one way ANOVA, *p* = < 0.0001. (**B**) Flow chart of model set-up, isolation of newly synthesized proteins, and mass spectrographic analysis carried out in this study. (**C**) Characterization of identified newly synthesized proteins according to the NCBI database entries of each protein and displayed as pie chart. (**D**) Molecular function enrichment analysis was carried out using the PANTHER overrepresentation test. The numbers of proteins annotated with each molecular function was plotted as a bar chart with the color scale representing the significance of the enrichment of molecular function within the list.

In order to develop a model to investigate breast cancer metastasis in vitro, we chose MDA-MB-231s due to their aggressive, epithelial to mesenchymal phenotype and well-evidenced ability to migrate and invade in vitro. Additionally, MDA-MB-231 belong to the basal/TNBC sub-type and are thus estrogen receptor negative and known to express the EGF receptor [23]. To determine whether EGF would be an effective chemoattractant in our models of migration and invasion, a series of transwell migration experiments were carried out. The highest level of migration of MDA-MB-231 cells was observed when EGF was present only in the lower chamber, as evidenced in Figure 1A; demonstrating that EGF elicits a specific increase in directional migration. Our own observations are in accordance with those published in similar studies [27,28,29,30] and gives evidence to the selection of EGF as a trigger for the processes of migration and invasion in MDA-MB-23 cells.

Having determined 50 ng/mL EGF to be an appropriate chemoattractant for our breast cancer cell line, it was selected as the stimulus in transwell migration and invasion assays in our model. The workflow of this model is summarized in Figure 1B. To identify the newly synthesized proteins while breast cancer cells undergo migration and invasion, MDA-MB-231 breast cancer cells were grown in 2-D and 3-D culture as described in Materials and Methods. Cell cultures were serum and methionine starved then stimulated with EGF and supplied with AHA. After stimulation, a fluorescent TAMRA-alkyne was added which binds to the azide moiety of the AHA-tagged newly synthesized proteins. This allowed for isolation of the newly synthesized proteins using anti-TAMRA in an immunoprecipitation reaction, with the resultant product being proteins newly synthesized following stimulation to migrate and invade towards an EGF chemoattractant. Proteins in these samples were then identified using mass spectrometry analysis. To specifically identify proteins that were newly translated during EGF stimulation, a control experiment without the addition of EGF was conducted for both the 2-D and 3-D models. Proteins identified in the EGF omitted controls were then subtracted from their respective EGF stimulated protein lists.

We identified a total of 95 newly synthesized proteins potentially involved in the migration and invasion of MDA-MB-231 breast cancer cells towards EGF (Table S1). Characterization of the protein list according to NCBI Protein [31] database entries found that the list spans a wide array of functions within the cell, with the highest proportions being: protein modification (e.g., kinases, phosphatases, proteases; 20%), structural (19%), and calcium binding (19%) as summarized in Figure 1C. We then carried out molecular function enrichment analysis using the PANTHER overrepresentation test which revealed the most significantly overrepresented category was that of proteins involved in calcium ion binding (GO:0045296, expected:0.97, input: 9, fold enrichment: 9.31, *p*-value: 7.56 × 10^−7^ false discovery rate: 3.82 × 10^−4^, Figure 1D). This functional characterization showed that proteins newly synthesized in the processes of cell migration and invasion encompass a wide variety of functions and numerous essential processes in cell signaling and behavior, many of which are implicated in cancer progression. 

### 3.2. Identification and Verification of Annexin A2 as A Newly Synthesized Protein in EGF Stimulated Migration and Invasion

**Figure 2:**
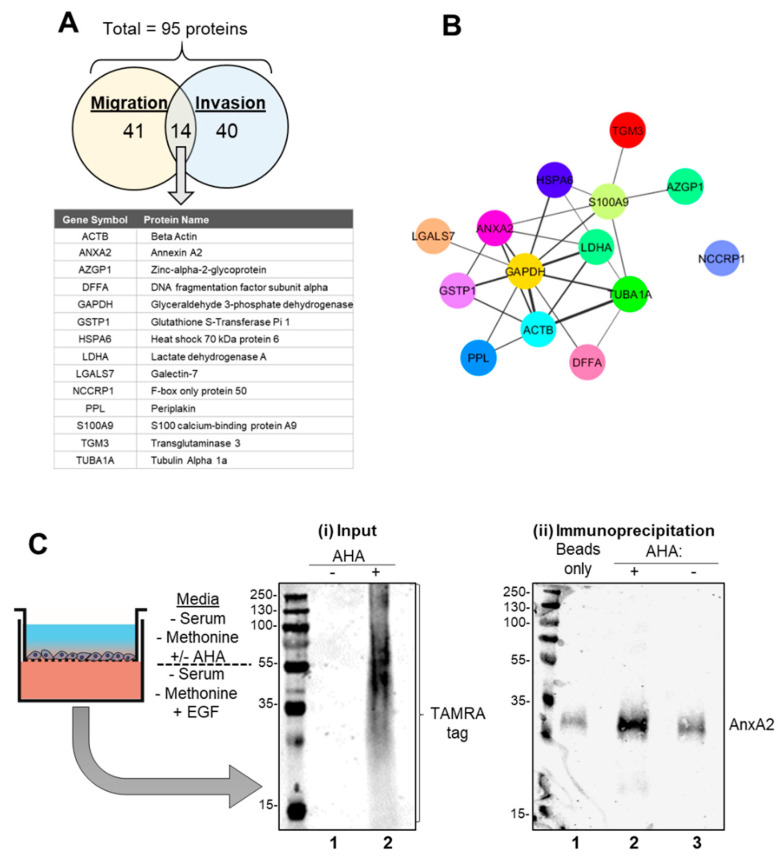
Annexin A2 is newly synthesized during the EGF stimulated migration and invasion of MDA-MB-231 cells. (**A**) 95 proteins in total were identified following mass spectrometry analysis and MASCOT peptide identification. Of these, 41 were found in the migration experiment only, 40 were found in the invasion experiment only and 14 were found to be common to both, listed here. (**B**) Protein-protein interactions between the 14 proteins found to be common to both the migration and invasion lists were assessed with String (http://string-db.org) [16] and visualized using the StringApp in Cytoscape set at 0.2 confidence level [17]. Edge line thickness denotes the confidence of association between protein nodes. (**C**) (**i**) Input: Western blot analysis of Click-It reaction lysate shows successful incorporation of the TAMRA tag into newly synthesized proteins when AHA was added. (Lane 1: Cell lysate of EGF migrating cells without AHA, Lane 2: Cell lysate of EGF migrating cells with AHA). (**ii**) Immunoprecipitation: Consequent TAMRA immunoprecipitation assay showed Annexin A2 pulldown following EGF induced cell migration as measured by SDS-PAGE and western blotting with anti-Annexin A2 antibody. (Lane 1: Beads only control, Lane 2: AHA+ lysate from lane 2 of (**i**), Lane 3 AHA- lysate from lane 1 of (**i**)).

Of the total 95 proteins identified as newly synthesized, 41 proteins were identified in the 2-dimensional culture migration experiment only, and 40 unique proteins were identified in the 3-dimensional culture invasion experiment only. When displayed in the Venn diagram in Figure 2A, 14 proteins were found to be common to both lists, meaning that they had been identified in both the 2-D migration and 3-D invasion models. Protein-protein interaction analysis of these 14 proteins using STRING [16] revealed an extensively linked network within the group, (with the exception of NCCRP1) as seen in Figure 2B. To identify key proteins within the identified network, each node was assessed for its degree of centrality, edge connectivity and whether it has been previously reported as important in breast cancer metastasis. Annexin A2, having satisfied these criteria and was chosen as a candidate protein to investigate how perturbing this elucidated network would affect breast cancer cell migration and invasion. Annexin A2 was also chosen as it was identified in both experiments, was relatively highly scored and due to its molecular function as a calcium ion binding protein [32], a gene ontology term found to be most significantly enriched (FDR = 3.82 × 10^−4^) following analysis of the total protein list. Annexin A2 is a member of the 12 protein annexin family, a group of proteins which have the ability to bind negatively charged membrane phospholipids in a calcium dependent manner [33,34]. In addition to the interactions illustrated in Figure 2B, Annexin A2 has proven links with a number of other identified proteins such as beta- actin [35] and tubulin [36]. Thus, it is highly probable that Annexin A2 is playing a central role in influencing this network, for example through its roles in actin polymerization [37] and cytoskeletal rearrangement [38,39]. In addition, due to the integral role and embryonic lethality of essential cytoskeletal proteins like beta actin [40], we believe there is more potential therapeutic value in investigating proteins that have a more regulatory, non-essential role, such as Annexin A2 [41]. In addition, Annexin A2 has a known clinical association with cancer [42] and has a role downstream of growth factor signaling pathways such as the IGF-IR [43] and the EGFR [44,45] pathways. This association was first established in hepatocellular carcinoma in 1990 [46] but to date, it has been found to be commonly overexpressed in multiple cancer types such as colorectal, breast, lung and pancreatic [42]. The amino acid sequence of Annexin A2 with the matched peptide sequences is shown in Figure S1 (Score: 169, Monoisotopic mass (Mr): 38808, Calculated pI: 7.57, Matches: 5, Sequences: 4, Coverage: 12%). The reported mass agrees with Annexin A2′s predicted molecular weight of 38 kDa. A representative MS/MS spectrum of one Annexin A2 peptide is also shown. Apart from Annexin A2 several other proteins of potential future interest were identified (Figure 2A,B). Intriguingly, a number of metabolism associated proteins, including GAPDH, LDHA, and GSTP1 were identified. Altered metabolism is a fundamental hallmark of cancer [47] and it has even been suggested that specific deregulations in the metabolism of metastasizing cells could be a specific therapeutic target [48].

Following identification of Annexin A2, we proceeded to validate it as a newly synthesized protein by replicating the initial migration experiment. This was done by performing a TAMRA immunoprecipitation (IP) of the Click-iT reaction lysate to isolate proteins that had incorporated the TAMRA tag then western blot analysis to show successful Annexin A2 pulldown (Figure 2C). Pull down of Annexin A2 is clearly increased in the AHA+ lysate sample (Panel (ii) lane 2) in comparison to the beads only (no antibody) control (Panel (ii), lane 1) and AHA- (non-TAMRA labeled) lysate control (Panel (ii), lane 3).This pulldown validates our approach and verifies our identification of Annexin A2 as a newly synthesized protein via mass spectrometry analysis.

### 3.3. Annexin A2 Expression is Increased in Estrogen Receptor Negative Breast Cancer Cells

**Figure 3:**
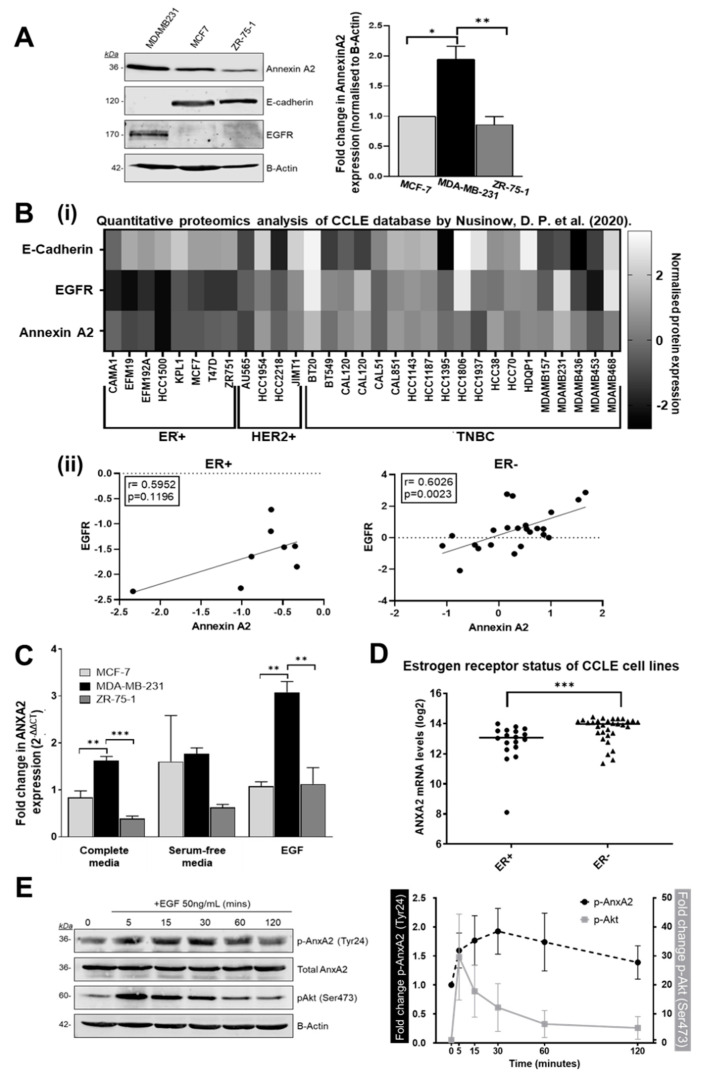
Annexin A2 expression is increased in ER negative breast cancer cells and undergoes transient phosphorylation following EGF stimulation. (**A**) Comparison of Annexin A2 expression in three breast cancer cell lines: MDA-MB-23, MCF-7 and ZR-75-1 as measured by SDS-PAGE and western blot. Protein expression was quantified by densitometric signal analysis on Image Studio software (LI-COR). Annexin A2 signal was normalized to beta-actin signal, then expressed in comparison to MCF-7 expression as 1. Data shown is mean +/− SEM for 3 individual experiments, measured by ANOVA with Bonferroni, *p* = 0.0034. (**B**) (**i**) Heatmap of Annexin A2, EGFR and E-Cadherin protein expression data for breast cancer cell lines from Nusinow, D. P. et al. (2020), stratified according to breast cancer subtype. Protein expression represented as normalized relative protein expression, as measured by quantitative proteomics. (ii) Scatterplots of the same data indicating the relationship between Annexin A2 and EGFR protein expression. A significant and strong positive correlation between Annexin A2 and EGFR expression is seen for ER- cell lines (Pearson’s correlation, r = 0.6026, *p* = 0.0023) (**C**) Gene expression of ANXA2 in three breast cancer cell lines: MDA-MB-231, MCF-7 and ZR-75-1. Expression was calculated using delta CT method and displayed as fold change (corrected RQ) in comparison to MCF-7 expression for each condition. (i.e., MCF-7 expression normalized to 1) differences between cell lines analyzed by ANOVA with Bonferroni. (**D**) Publicly available Affymetrix gene expression data for human breast cancer cell lines was obtained from the CCLE. Cell lines were categorized according to ER expression status and difference in ANXA2 expression between the 2 groups was measured using Mann–Whitney U test, *p* = 0.0008 (**E**) Western blot showing the effect of EGF stimulation on AnxA2 expression and phosphorylation in MDA-MB-231 cells. After 4 h of serum starvation, cells were treated with 50 ng/mL EGF. Representative blot and corresponding time course graph showing the increase in AnxA2 phosphorylation at Tyr24 followed by decrease after 30 min. Densitometry signal normalized as phosho-AnxA2/total AnxA2 and phospho-Akt/total beta-actin. Data points displayed as mean of 3 individual experiments ±SEM.

Following identification and verification of Annexin A2 as a protein newly synthesized in EGF mediated MDA-MB-231 cell migration and invasion, we next examined its expression profile across a number of breast cancer cell lines. Western blotting analysis of cell protein content shows an increased expression of Annexin A2 in the estrogen receptor negative (ER-), basal/TNBC cell line MDA-MB-231 when compared to the estrogen receptor positive (ER+), luminal cell lines MCF-7 and ZR-75-1 (both ER+/PR+) (Figure 3A). Expression of Annexin A2 appears to inversely correlate with expression of E-Cadherin, the loss of which is a known marker of epithelial to mesenchymal transition. To further support these findings, we analyzed data from D. P. Nusinow et al. (2020) [19] to generate a protein expression heatmap (Figure 3B) showing how the expression of Annexin A2, EGFR, and E-Cadherin vary across a larger sample of cell lines, according to breast cancer subtype classification. From this it is evident that there is an increase in both Annexin A2 and EGFR expression in the ER- (HER2 and TNBC) cell lines when compared to ER+ cell lines (further depicted in Figure S2). This supports and is in agreement with our conclusions drawn from our western blot data (Figure 3A). Interestingly, we do not see an association between the loss of E-Cadherin and elevated Annexin A2 when we query this larger cohort of cell lines, thus the trend observed in Figure 3A may be attributed to individual characteristics of the three cell lines tested. When we consider the potential relationship between Annexin A2 and EGFR expression, our analysis shows that in ER- cell lines, there is a strong positive and significant correlation between the expression of these two proteins (Pearson’s correlation, r = 0.6026, *p* = 0.0023). Importantly, and in support of our hypothesis, we do not see this significant correlation in ER+ cell lines (r = 0.5952, *p* = 0.1196).

Next, we examined Annexin A2 mRNA expression using RT-qPCR in breast cancer cell lines, under normal, serum starved and EGF stimulated conditions. Again, we observed Annexin A2 expression to be increased in ER- cells in comparison to ER+ cell lines under all culture conditions (Figure 3C). In particular, we found that following 4 h of 50 ng/mL EGF stimulation ANXA2 is even more highly expressed in MDA-MB-231s when compared to the other ER+ cell lines. This suggests that the increased expression of ANXA2 in response to EGF is specific to the ER-, EGFR expressing MDA-MB-231 cell line. We then utilized the available data within the Cancer Cell Line Encyclopedia (CCLE) [21] to further probe the relationship between ER status and AnxA2 expression. The CCLE is a large, publicly available data repository which compiles gene expression, chromosomal copy number and sequencing data from 947 human cancer cell lines, spanning 36 tumor types [21]. Breast cancer cell lines with ANXA2 expression data available (*n* = 49) were first categorized according to positive or negative ER status according to previously published molecular characterization studies [22,23]. This stratification revealed 18 of the available cell lines to be ER+ and 31 to be ER-. Comparing levels of ANXA2 mRNA expression between ER+ and ER- cell lines, as measured by Affymetrix U133 plus 2.0 arrays, showed ER- lines exhibit significantly higher expression of ANXA2 (*p* = 0.0008) as shown in Figure 3D. The cell lines analyzed and their respective ANXA2 expression values are outlined in Figure S2. These results show that expression of Annexin A2 in breast cancer is strongly influenced by estrogen receptor status and that its overexpression in breast cancer may be restricted to the ER- subtype. ER- negative breast cancers include both TNBC and HER-2 over expressing subtypes, both of which are more aggressive cancers with a poorer prognosis [49].

As the synthesis of Annexin A2 identified in our initial experiment was triggered by stimulation with EGF, we investigated the effect of EGF on the regulation of Annexin A2 (Figure 3E). To control our experiment, we examined the p-Akt response, which was used as a read-out of our serum starvation and EGF stimulation. We found that stimulation of MDA-MB-231 cells with EGF resulted in an increase in phosphorylation of Annexin A2 on Tyrosine 24 (Tyr24) which reached a peak phosphorylation at 30 min, before decreasing towards, initial, pre-stimulation levels over 120 min (Figure 3E). This post-translational modification allows Annexin A2 to bind to S100A10 (also known as p11) to form the AnxA2/p11 heterotetramer which then is translocated to the extracellular cell surface [50,51]. This modulation is of particular relevance in cancer phenotype as once on the cell surface, AnxA2/p11 promotes the conversion of plasminogen to active plasmin, a protease which plays a role in extracellular matrix degradation and activation of matrix metalloproteases, critical steps in cancer cell metastasis [34,52]. In fact, Tyr24 phosphorylation has been shown to be critical for the invasive potential of multi-drug resistant breast cancer cells [53] and is proposed to modulate epithelial to mesenchymal transition in a number of cancer types, including pancreatic [54], cervical [55], colon [56] and lung [57].

### 3.4. Annexin A2 is Required for Cell Proliferation, Wound Healing and EGF Directed Cell Migration of ER Negative Breast Cancer Cells

**Figure 4:**
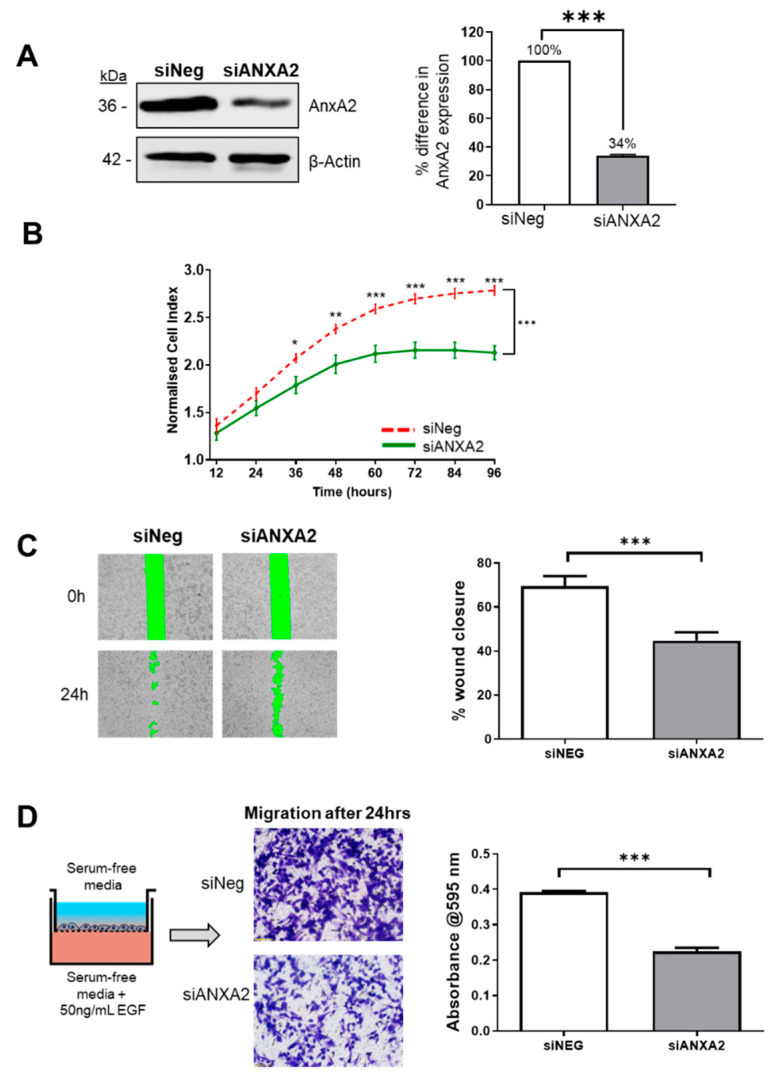
siRNA mediated knockdown of AnxA2 inhibits the proliferation and migration of MDA-MB-231 cells. (**A**) MDA-MB-231 cells were transfected with a small interference oligonucleotide against ANXA2 [10 nM] for 72 h, using the Neon Transfection system. Representative blot showing the efficiency of ANXA2 knockdown (KD) after 72 h and densitometry analysis of Annexin A2 protein bands normalized against beta-actin loading control and expressed as a percentage of AnxA2 level in negative control, measured using Image Studio software. Data displayed as mean +/− SEM for three individual experiments. (**B**) Reduction of Annexin A2 expression in MDA-MB-231 cells significantly inhibits cell proliferation as measured by real time change in cell impedance. KD and negative control cells were seeded in duplicate into E-plate wells and the rate of proliferation was measured in real-time using the xCELLigence system. Proliferation of KD cells (green) was compared to the proliferation of negative control wells (red) over the course of 96 h. Data displayed as mean cell index +/− SEM for three individual experiments. (**C**) Reduction of Annexin A2 expression in MDA-MB-231 cells significantly inhibits cell migration as measured by wound healing assay. Knockdown and negative control cells were plated into dishes containing ibidi Culture Inserts. After 72 h, the inserts were removed to generate a cell free wound. The green zone indicates the wound size as measured at 0 h and 24 h post-removal of insert. Data displayed as percent wound closure for 3 individual experiments (6 fields of view each) +/− SEM, *p* = 0.0002. (**D**) Reduction of Annexin A2 expression significantly inhibits EGF directed cell migration as measured with transwell migration chambers. Cells were plated onto the upper chamber of transwell membrane and allowed to migrate towards 50 ng/mL EGF chemoattractant for 24 h. Cells were removed from the upper chamber, leaving only those that had migrated to the lower chamber. Migrated cells were stained with crystal violet and imaged using an inverted light microscope. Crystal violet stain was dissolved, and intensity measured as absorbance at 595 nm in triplicate. Data displayed as mean absorbance values for 3 individual experiments +/− SEM, *p* < 0.0001.

Following evidence of Annexin A2′s involvement in the migration and invasion in our ER negative cell model, we next investigated its influence on the cancer phenotype with a series of functional assays. AnxA2 expression was suppressed in MDA-MB-231 cells using siRNA specific to AnxA2 mRNA transcript (Table S2). Figure 4A shows a representative blot of knockdown efficiency, showing reduced level of Annexin A2 protein in cells 72 h following transfection. Firstly, using a real time cell analysis platform (xCELLigence) the effect on the growth and proliferation of MDA-MB-231 cells was monitored by measuring cell index every 15 min over the course of 96 h. As shown in Figure 4B, the depletion of AnxA2 significantly reduced the proliferation of MDA-MB-231 cells when compared to control cells. Next, the effect of AnxA2 knockdown on cell migration was then assessed using two independent assays. We first used wound healing assays which revealed knockdown cells to have a significantly decreased ability to close the wound after 24 h (Figure 4C). This was followed by transwell chamber experiments, which specifically tested the effect on the directional migration and the response to an EGF chemoattractant. In this assay, MDA-MB-231 cells were plated in serum-free media on the upper chamber of the transwell and allowed to migrate towards the bottom chamber, containing serum free media supplemented with 50 ng/mL EGF. We found that ANXA2 knockdown cells were significantly reduced in their ability to migrate towards an EGF gradient (Figure 4D). These results are consistent with previous reports of Annexin A2 being a positive regulator of wound healing in MDA-MB-231 cells [44,58]. However, they also point towards the role of Annexin A2 in promoting specific EGF directed migration. In addition, we have shown that Annexin A2 knockdown inhibits the proliferative ability of the ER- MDA-MB-231 cells. These functional results were confirmed by replication of each assay using another clone targeting ANXA2 (Figure S4). Taken together, this data strongly supports the hypothesis that Annexin A2 promotes cell proliferation, wound healing and cell migration, all critical processes which support the malignant phenotype of ER- breast cancer cells in vitro.

### 3.5. Gene Expression Analysis of Breast Cancer Tissue Shows ANXA2 is Specifically Upregulated in ER Negative Breast Cancer and Correlates with Rates of Metastasis

**Figure 5:**
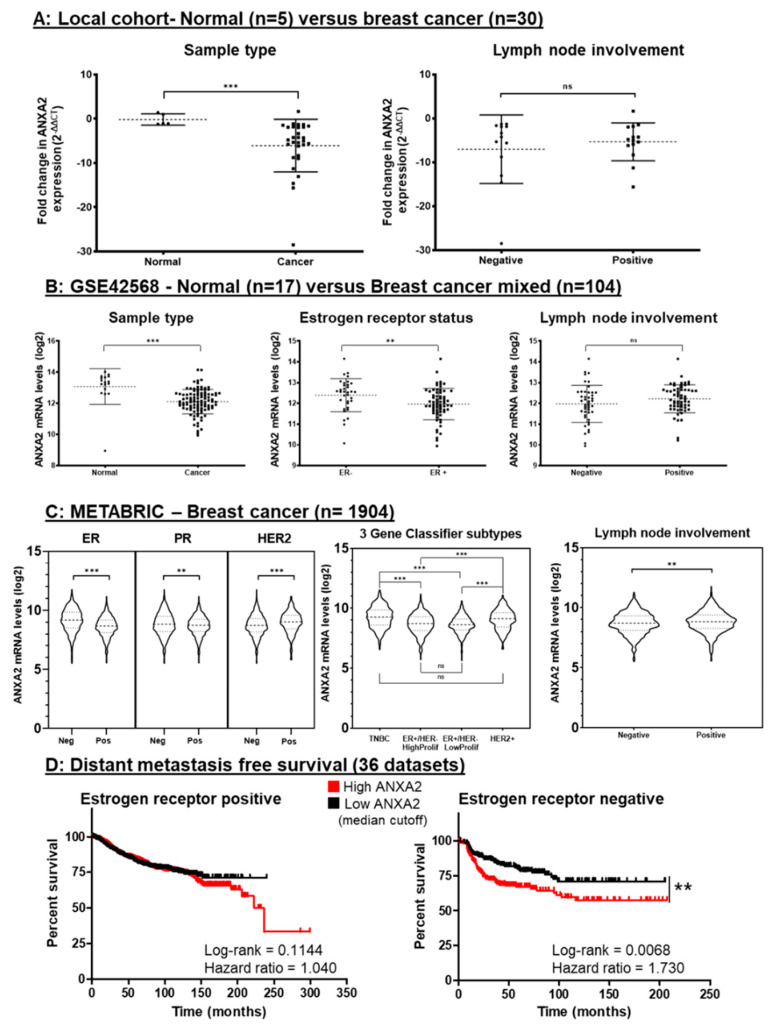
ANXA2 expression is associated with and is a prognostic indicator in ER negative breast cancer. (**A**) Analysis of ANXA2 expression using qRT-PCR of normal (*n* = 5) and breast cancer (*n* = 30) tissue samples. Analysis showed ANXA2 is significantly upregulated in normal tissue versus cancer tissue (*p* = 0.0002). Gene expression represented as fold change from normal closest to mean. Horizontal bars represent the mean and SD of fold change. Statistical difference between groups tested using Mann Whitney U test. (**B**) Analysis of ANXA2 mRNA expression in GSE42568 showing ANXA2 is upregulated in normal tissue versus cancer tissue, (*p* < 0.0001), in ER- breast cancer versus ER+ breast cancer (*p* = 0.0094) and in patients with nodal positive disease (*p* = 0.1121). Horizontal bars represent the mean and SD gene expression levels. Statistical difference between groups was measured using Student’s t-test. (**C**) Analysis of ANXA2 mRNA expression of the METABRIC cohort showing upregulation of ANXA2 in ER negative (*p* < 0.0001), PR positive (*p* = 0.0013) HER2 positive (*p* < 0.0001) and in nodal positive (*p* = 0.0050) breast cancer patients. Using the 3 gene classifier (42) to stratify patients shows TNBC and HER2+ subtypes to have significantly higher expression of ANXA2 than both ER+/HER2- subtypes. Statistical difference between groups was calculated using Mann–Whitney U test. (**D**) Kaplan–Meier curves comparing the DMFS of patients with high ANXA2 expression (red) versus low ANXA2 expression (black) as determined by median expression value cut-off. Curves were analyzed using univariate log-rank tests. Expression of ANXA2 was found to not have a significant effect in ER+ patients whereas it did significantly increase probability of distant metastasis for ER- patients (*p* = 0.0068).

Due to our accumulating evidence of Annexin A2′s importance in breast cancer progression in our in vitro cell models, we next investigated its expression in breast cancer patient tissue. RT-qPCR was used to analyze the level of ANXA2 mRNA in core biopsy tissue samples (normal = 5, cancer = 30). Surprisingly, despite several reports in the literature that ANXA2 is upregulated in breast cancer [42,59,60], we observed that expression of ANXA2 is significantly down regulated (*p* = 0.0002, Mann Whitney U test) in breast cancer tissue in comparison to pathologically normal breast tissue (Figure 5A). When ANXA2 expression levels were stratified according to nodal involvement—an indicator of breast cancer disease progression—we found a trend of increased ANXA2 expression in those who had nodal positive disease, but this was not statistically significant (*p* = 0.8674, Mann Whitney U test). Due to the sample size of this local cohort, the expression of ANXA2 according to receptor status could not be carried out reliably (ER+ = 24, ER- = 2, see Table S3). Thus, to add power to our investigation and to further interrogate the relationship between ER status and ANXA2 expression we then extended our study to publicly available gene expression datasets.

The NCBI GEO database is a public functional genomics data repository through which anonymized gene expression and corresponding clinical attributes can be accessed and analyzed. The dataset GSE42568 was chosen to closely reflect our local experimental cohort as it also contained gene expression data from normal (*n* = 17) and breast cancer (*n* = 104) tissue from Irish patients. Fold change of ANXA2 expression was measured as log2 using Affymetrix microarrays and probe set 201590_x_at and differences between groups was measured using Student’s t-test. Analysis of this data set (Figure 5B) also showed a significant down regulation in ANXA2 in breast tumor tissue compared to normal breast tissue (*p* < 0.0001, Mann–Whitney U). Further, by categorizing the GSE42568 cohort according to ER expression, we see that ANXA2 expression is significantly upregulated in those with ER negative breast cancer tissue (*p* = 0.0094, Student’s t-test), which is consistent with our cell line data. Reflecting the results seen in our RT-qPCR local cohort, ANXA2 appears to be slightly increased in those with nodal progression in GSE42568 (*p* = 0.1121, Student’s t-test). The expression status of other receptors such as progesterone receptor (PR) and HER-2 were not recorded for this dataset (Table S4). To further verify the relationship between Annexin A2 and ER- breast cancer tissue, we interrogated available quantitative proteomic data from S. Tyanova et al. (2016) [25]. Through this we found, in support of our argument, that Annexin A2 expression at the level of the protein is significantly higher in ER- breast cancer tissue (*p* = 0.0185, Student’s t-test) (Figure S5).

These trends are further evidenced by querying the METABRIC (Molecular Taxonomy of Breast Cancer International Consortium) cohort for ANXA2 expression. METABRIC is a publicly available database containing genomic and clinical data for over 2000 breast cancer patients [61]. As illustrated in Figure 5C, again, ER negative patients appear to have a higher expression of ANXA2 (*p* < 0.0001, Mann–Whitney U). When considering expression of other receptors, it can be seen that PR negative patients have a slight increase in ANXA2 expression overall (*p* = 0.0037, Mann–Whitney U), whereas HER-2 positive patients have increased ANXA2 expression compared to those who do not have HER-2 overexpression (*p* < 0.0001, Mann–Whitney U). The 3-gene classifier postulated by Haibe-Kains B et al. [62] allows for the stratification of tumor samples into four main clinical subtypes: ER+ high proliferation, ER+ low proliferation, TNBC (denoted as ER-/HER2- in METABRIC) and HER2+. Comparing ANXA2 expression between these subtypes reveals both ER positive subtypes to have a significantly lower expression of ANXA2 than their ER negative counterparts. (TNBC vs. ER+hi prolif, *p* < 0.0001; TNBC- vs. HER2+, *p* = 0.2737; TNBC vs. ER+ lo prolif, *p* < 0.0001; ER+ hi prolif vs. HER2+, *p* < 0.0001; ER+ hi prolif vs. ER+ lo prolif, *p* = 0.4909; HER2+ vs. ER+lo prolif, *p* < 0.0001, Kruskal-Wallis with Dunns adjustment). Once more, analysis of this large breast cancer cohort appears to link ANXA2 expression with ER negative breast cancer, encompassing both the aggressive triple negative and HER-2 overexpressing subtypes. In addition, those with a positive nodal status also have an increased expression of ANXA2 when compared to those without nodal involvement, regardless of receptor status (*p* = 0.0050, Mann–Whitney U).

Taking into account the specificity we see of ANXA2 overexpression in ER-negative breast cancer and the potential influence ANXA2 has on disease progression in terms of lymph node involvement, we then wanted to ascertain the effect ANXA2 expression has on prognosis for breast cancer patients. Gene expression and survival data sets were downloaded from KMPlot.com and categorized according to ER expression (derived from gene expression data) [26]. Kaplan–Meier plots and log-rank tests were used to compare the distant metastasis free survival (DMFS) rates of patients stratified according to high or low ANXA2 expression, illustrated in Figure 5D. Using a median value as high/low cut off for ANXA2 expression, we found that for ER positive patients (*n* = 1395), ANXA2 expression had no significant effect on DMFS. Contrastingly, in patients with ER negative breast cancer (*n* = 351), high ANXA2 expression had a significant detrimental effect on DMFS, with a hazard ratio showing that those with high expression were almost 2 times more likely to develop distant metastasis (*p* = 0.0068, HR = 1.730). These results indicate that ANXA2 expression is correlated with poor clinical outcome, specifically distant metastasis for only the ER negative grouping of breast cancer patients.

## 4. Discussion

In this study, we utilized well-established transwell chamber assays, in combination with the BONCAT method and mass spectrometry to identify the nascent proteome that is synthesized when breast cancer cells undergo migration and invasion towards an EGF chemoattractant. This is an innovative method to assess the global proteomic changes that occur during these processes of cancer progression using an in vitro model. This experimental set-up allowed for a highly specific and controlled insight into the early translational events involved in the metastatic process in a model of aggressive ER negative breast cancer. Functional analysis of the proteins identified in this procedure showed that a wide range of cellular functions were regulated by the chemotactic EGF stimulation. Of these functions, gene ontology enrichment analysis revealed calcium ion binding to be the most significantly overrepresented GO term within our list. Calcium signaling has well-established links to processes of cancer progression such as proliferation, apoptosis and migration and invasion [63,64]. Furthermore, calcium flux has an intrinsic role in the breast due to lactation and the expression of several calcium pumps, involved in milk production, have been implicated in breast cancer [65]. Altered calcium homeostasis has been observed within breast cancer, however, it is currently unclear whether the dysregulation of calcium binding proteins like Annexin A2 are a cause or a consequence of this imbalance [64]. Despite this, it is known that Ca^2+^ level within the cell regulates Annexin A2′s affinity for its binding partners and cellular components such as the plasma membrane and the actin cytoskeleton [51,66,67]. Apart from Annexin A2, calcium binding proteins such as the S100 family (several of which were identified in our screen, Table S1) have been thoroughly investigated in the context of cancer, as reviewed by Bresnick et al. [68] and these, along with Annexin A2, have the potential to be utilized as prognostic or therapeutic targets in breast cancer [69]. As well as successfully identifying Annexin A2 as a mediator of breast cancer cell migration and invasion, our screen has provided an extensive list of proteins potentially involved in breast cancer metastasis which require further investigation (Table S1). This valuable information can guide our and others’ future research into deciphering the mechanics of cancer progression.

As migration and invasion constitute integral mechanisms in the development of cancer metastasis, the proteins identified by our model and mass spectrometry screen potentially have a role in cancer progression. We thus tested the value of our experimental design by conducting a focused study on Annexin A2—a protein identified in both our migration and invasion experiments. Annexin A2 is a calcium ion regulated membrane binding protein [67] found to play a role in a wide range of cellular processes, from endo- and exo- cytosis to proliferation and apoptosis [51]. One of its best-known functions is its hand in the proteolytic cascade, triggered by the activation of plasmin. The plasminogen activation system is vital for the tissue remodeling required for wound healing. This process however, has also been associated with cancer progression [70]. AnxA2 promotes the conversion of the inactive pre-protein plasminogen into its active version, the serine protease called plasmin. AnxA2 does this by binding both tissue plasminogen activator (tPA) and plasminogen thus bringing enzyme and substrate together spatially and significantly increasing the rate of conversion [71]. This fibrinolytic process produces proteases with the ability to degrade extracellular matrix (ECM) proteins and further activate matrix metallo-proteases (MMPs). When dysregulated, this degradation ability greatly accelerates the progression of cancer by giving the tumor a greater chance to invade through the ECM and metastasize to other locations in the body [60]. Annexin A2 has been proposed as a potential biomarker and therapeutic target for aggressive and metastatic cancers [72,73]. A recent study by Shen et al. even postulated the use of phosphorylated Annexin A2 for the selective imaging of solid tumors within the clinic [74]. Studies by Sharma et al. [58] and Yeatman et al. [75] have actually shown that the expression of Annexin A2 is higher in metastatic cancer cell lines versus non-metastatic cancer cells. The recent authoritative review by Sharma discusses these studies and similar findings in a number of cancer types and concluded by proposing Annexin A2 to be a universal signature of aggressive and metastatic cancer [72]. Our approach has added to the consensus that Annexin A2 plays an important role in the progression of breast cancer, suggesting that stimulus to migrate and invade with an EGF chemoattractant actually induces the nascent translation of Annexin A2.

The patterns of higher Annexin A2 expression at both a protein and gene level, as demonstrated using both western blotting and RT-qPCR methods (Figure 3A,C), as well as through interrogating independent genomic and proteomic data repositories (Figure 3B,C) suggested the importance of Annexin A2 expression in ER negative breast cancer. Thus, we next assessed the functional consequences of decreasing Annexin A2 expression using RNA interference techniques within an ER negative breast cancer model. Knockdown of Annexin A2 attenuated many of the traditional hallmarks of cancer exhibited by MDA-MB-231 cells. Cell growth and proliferation was diminished, adding to the evidence of Annexin A2′s role in cell division, as reported in a number of experimental models [76,77]. Cell migration, as measured by both wound healing assay and EGF-induced directional migration, was significantly diminished upon reduction of Annexin A2 expression. As discussed earlier, cancer cell motility and chemotactic response to factors found within the tumor microenvironment are pillars of metastatic progression. Annexin A2 has been implicated in cell motility due to its role in binding actin filaments, regulating filament polymerization, and cytoskeletal organization [35,36,37]. In addition, it has been shown to recruit Rho GTPases, cofilin and other motility associated effector proteins [38,39,45,78,79] This role of Annexin A2 in promoting breast cancer cell migration equates with results from previous research [44,80,81] but to our knowledge is the first instance to show the specific reaction to an EGF gradient. To further validate the clinically relevant role of Annexin A2 in metastasis, future investigations are needed. Studies using Matrigel plug assays [82] and xenograft models [83] have indicated the potential of targeting Annexin A2 in angiogenesis and tumor growth in vivo. However, various measurements of invasion such as 3-D spheroid formation and gelatin zymography, in combination with physiologically relevant, spontaneous metastatic mouse models (such as those used in [84]) are essential for a fully comprehensive insight into Annexin A2′s role in the metastatic cascade.

A link with EGF signaling could be contributed to the interaction between Annexin A2 and the EGF receptor (EGFR). Our results (Figure 3B(ii)) indicate a strong correlation between the expression of EGFR and Annexin A2 in ER negative cell line models. However, within the literature, this relationship is riddled with conflicting reports with some data showing that reduction of Annexin A2 dampens the downstream activation of the EGFR pathway [44]; while others report that inhibition of Annexin A2 expression enhanced both EGF induced cell migration and downstream activation of JNK and Akt in mouse models [45]. Indeed, our results (Figure 3D) and others [85], show that EGF stimulation appears to transiently increase Annexin A2 phosphorylation at Tyr24. This phosphorylation is likely caused by EGFR mediated activation of Src kinase, of which Annexin A2 is a known substrate [44]. Current literature suggest that this phosphorylation promotes Annexin A2 translocation to cell and exosomal surfaces where it can enact its pro-metastatic functions by bridging proteins together to activate ECM remodeling enzymes and even attract pro-tumorigenic macrophages into the TME [54,58,81,86,87,88]. Recent work by Y Fan et al., showed that the reduction in migration and invasion attributed to Annexin A2 suppression can be rescued by the exogenous expression of a phospho-mimic-ANXA2 but not by a phospho-null-ANXA2, highlighting the importance of the phosphorylation we have observed [53]. Furthermore, a number of studies have linked growth factor mediated phosphorylation of Annexin A2 to the promotion of EMT in a variety cancer types [55,56,57,81]. Although the methods used for assessing EMT vary, many come to the consensus that Annexin A2 plays a role in triggering the mesenchymal and migratory phenotype in cancer cells via the transcriptional program of EMT. This agrees with our observations of Annexin A2 suppression diminishing the migratory capacity of breast cancer cells (Figure 4C,D) and potentially correlating with loss of EMT marker, E-Cadherin, in specific cases (Figure 3A). Despite the increase in Annexin A2 phosphorylation and the nascent translation proposed by our mass spectrometry identification (Figure 3D), the level of total Annexin A2 within the cell remains constant when measured by western blot. This is similar to results seen by Maji et al. in which exosomal Annexin A2 was seen to increase in a progressively metastatic breast cancer cell line model, despite seeing no change when the whole cell lysate is analyzed [87]. This could suggest that the total cellular pool outweighs the newly synthesized pool of Annexin A2 that is translated upon directional EGF stimulation, thus masking any potential increase in protein levels. This is supported by reports of Annexin A2 having a long half-life (40–50 h) [89,90,91] and by our own findings that suggest that it is only 72 h post transfection with siRNA against ANXA2 that we see the knockdown and maintenance of its knockdown beyond 35% (data not shown). Further examination is ongoing to ascertain whether Annexin A2 is newly synthesized during this process to replace the protein lost to cell surface translocation. Exosomal or secreted Annexin A2 has been reported to promote breast cancer metastasis and angiogenesis in a number of studies [58,82,87] and has even been proposed as a serum based biomarker for breast cancer detection [82,92]. Taken together, this suggests that Annexin A2 is involved in processes that promote breast cancer disease progression and this could be particularly true for tumors driven by EGFR overexpression. This is further compacted by our findings of a significant correlation between Annexin A2 and EGFR expression and by the observation that breast cancers found to have EGFR overexpression are more commonly ER negative than ER positive [93].

Having established the importance of Annexin A2 expression in maintaining proliferation, wound healing, and migration within our in vitro model (Figure 3), we next moved to investigate its expression in patient tissue. Interestingly, we found that when unstratified breast cancer samples were compared to normal breast tissue, Annexin A2 expression was significantly lower in the cancer tissue. This finding was validated in the larger GSE4825 cohort. This is in contrast to immunohistochemical analysis by Sharma et al. [58] which showed Annexin A2 to be undetectable in normal breast epithelia but strongly and consistently expressed in invasive breast cancer tissue. This discrepancy could be explained by a differential level of translational control between mRNA to protein. However, this requires further investigation as our result poses a challenge to the current general consensus that Annexin A2 is upregulated in breast cancer compared to pathologically normal tissue [42,59,60].

The stratification of breast cancer patients according to their ER expression status (GSE42568, METABRIC and S. Tyanova, et al. (2016) cohorts) revealed that the trend seen in cell lines also applies to human breast cancer tissue samples. ER negative breast cancer encompasses both the triple negative and HER-2 overexpressing subtypes [49]. These are often more aggressive and have fewer treatment strategies in comparison with the more common ER positive breast cancers. Women diagnosed with ER negative tumors are usually diagnosed at an earlier age and experience a higher mortality rate [94]. With the exception of trastuzumab’s unmatched beneficial effect for HER-2 positive patients [95], traditionally, toxic and unspecific chemotherapy is used to treat ER negative tumors. In addition, a caveat to the success of trastuzumab for HER-2 positive breast cancer is that a large majority of those treated develop resistance over time [96]. This highlights the need for targeted therapies which can mimic the wide reaching success of anti-estrogens such as tamoxifen for ER positive cancers [97]. The large sample size of the METABRIC analysis also revealed Annexin A2 to be upregulated in HER2 positive breast cancer, contradicting what has been previously published [44] and suggesting the value of investigating Annexin A2 in breast cancer is not limited to the triple negative subtypes. The potential of Annexin A2 as a therapeutic target or marker of aggressiveness in ER negative breast cancer is further solidified by its links to disease progression and metastasis. Analysis of our local tissue cohort (RT-qPCR), GSE42568, and the METABRIC (Affymetrix) cohort all show that Annexin A2 expression is higher in patients with lymph node involvement (Figure 5A–C). Due to the lymphatic drainage of the breast tissue, the lymph nodes are often the first location of metastatic spread. Presence of metastasized tumor cells in the axillary lymph nodes is one of the strongest prognostic indicators in breast cancer [98] and as a result, lymph node status is a parameter used to stage the disease [99]. The trend of higher ANXA2 expression in patients with lymph node involvement in our local cohort and the larger GSE42568, is not statistically significant, however, this could be attributed to the fact that only a small proportion of patients in these cohorts are ER negative (Tables S3 and S4), thus masking the possible effect of ANXA2 expression on disease progression. Annexin A2 is again associated with metastatic progression of disease when we show that risk of distant metastasis is significantly higher in ER negative breast cancer patients with high expression of ANXA2. This statistically significant result does not hold true for ER positive patients, providing evidence that the potential of targeting Annexin A2 holds promise for the more aggressive and harder to treat ER negative breast cancers.

## 5. Conclusions

Collectively, we have developed a novel approach to assess the changes that occur in the nascent proteome when cancer cells undergo stimulation to carry out the pro-metastatic functions of invasion and migration. We have proven the validity and usefulness of this approach by demonstrating how Annexin A2, one of the elucidated proteins, plays a pivotal role in maintaining the malignant phenotype of estrogen receptor negative breast cancer. The variety of experimental techniques and independent transcriptomic and proteomic datasets encompassing both cell line models and patient tissue in this study provides an emphatic argument that Annexin A2 plays a key role in ER negative breast cancer. Our work has used innovative models to add substantial weight to the findings by others, linking Annexin A2 to breast cancer. This study provides novel insight into the potential mechanisms of metastasis in ER negative breast cancer. Further validation is needed to build upon this study and truly assess the viability of targeting Annexin A2 to prevent cancer metastasis in vivo. Ultimately, this knowledge suggests that targeting Annexin A2 merits investigation as a novel therapeutic strategy in treating the pharmacologically challenging ER negative subtype, and this may help prevent breast cancer progression to the later metastatic stages of disease.

## Supplementary Materials

The following are available online at https://www.mdpi.com/2073-4409/9/7/1582/s1. Figure S1: (A) Score and sequence coverage of Annexin A2 from mass spectrometry and MASCOT server results (B) representative mass spectrum of AnxA2 peptide Figure S2: Proteomic expression data for CCLE cell lines from Nusinow, D. P. et al. (2020) Figure S3: Publicly available Affymetrix gene expression data for human breast cancer cell lines. Figure S4: Additional functional assays with second siRNA clone on MDA-MB-231 cells. Figure S5: Proteomic expression data for breast cancer tissue samples from S. Tyanova, et al. (2016). Figure S6, S7, and S8: full, uncropped images of western blots in manuscript. Table S1: List of newly synthesised proteins identified in 2D migration experiment. Table S2: List of newly synthesised proteins identified in 3D invasion experiment. Table S3: Sequences of the small interfering RNA used for ANXA2 knockdown. Table S4: Clinical information of local breast cancer cohort (*n* = 30) Table S5. Clinical information of GSE42568 breast cancer cohort (*n* = 104).
