# Supplementary material for: Expression of Annexin A2 Promotes Cancer Progression in Estrogen Receptor Negative Breast Cancers
Source: Cells. 2020 Jun 30;9(7):1582. doi: 10.3390/cells9071582 (PMC7407301; doi:10.3390/cells9071582)
Supplement: Supplementary file 1 [file cells-09-01582-s001.pdf]

Supplementary information

Expression of Annexin A2 promotes progression in estrogen receptor negative breast cancer

Amira F. Mahdi[1 2], Beatrice Malacrida[1 2], Joanne Nolan[1 2], Mary E. McCumiskey[3 4], Anne B. Merrigan[4], Ashish Lal[4], Shona Tormey[4], Aoife J. Lowery[5], Kieran McGourty[2 6], Patrick A. Kiely*[1 2]

[1]Graduate Entry Medical School, University of Limerick, Limerick, Ireland; amira.mahdi@ul.ie (A.F.M); b.malacrida@qmul.ac.uk (B.M); joanne.nolan@ul.ie (J.N); patrick.kiely@ul.ie (P.A.K)
[2]Health Research Institute, University of Limerick and Bernal Institute, University of Limerick, Ireland; kieran.mcgourty@ul.ie (K.MG)
[3]Stokes Laboratories, Bernal Institute, University of Limerick, Limerick; mary.mccumiskey@ul.ie (M.E.MC)
[4]Department of Surgery, University Hospital Limerick, Limerick, Ireland; ashish.lal@hse.ie (A.L) anne.merrigan@hse.ie (A.B.M); shona.tormey@hse.ie (S.T)
[5]Lambe Institute for Translational Research, National University of Ireland Galway, Ireland; aoife.lowery@nuigalway.ie (A.J.L)
[6]Department of Chemical Sciences, University of Limerick, Limerick, Ireland.

*** Correspondence: patrick.kiely@ul.ie ; Tel.: +353-61-202811**

Figure S1

(A)

Score	Mass	Matches	Sequences	Coverage
169	38808	5	4	12%

(B)

1	MSTVHEILCK	LSLEGDHSTP	PSAYGSVKAY	TNFDAERDAL	NIETAIKTKG
51	VDEVTIVNIL	TNRSNAQRQD	IAFAYQRRTK	KELASALKSA	LSGHLETVIL
101	GLLKTPAQYD	ASELKASMKG	LGTDEDSLIE	IICSRTNQEL	QEINRVYKEM
151	YKTDLEKDII	SDTSGDFRKL	MVALAKGRRA	EDGSVIDYEL	IDQDARDLYD
201	AGVKRKGTDV	PKWISIMTER	SVPHLQKVFD	RYKSYSPYDM	LESIRKEVKG
251	DLENAFLNLV	QCIQNKPLYF	ADRLYDSMKG	KGTRDKVLIR	IMVSRSEVDM
301	LKIRSEFKRK	YGKSLYYYIQ	QDTKGDYQKA	LLYLCGGDD	

(C)

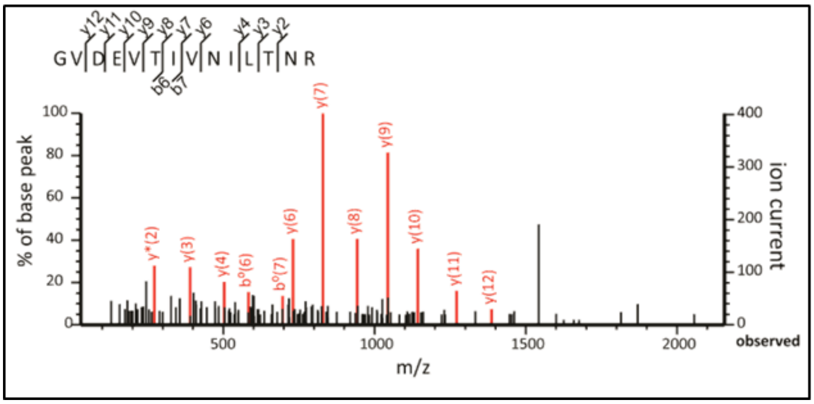

Figure S1: Mass spectrometry identification of Annexin A2 as a protein newly synthesised during the EGF stimulated migration and invasion of MDA-MB-231 cells.
(A& B) Scoring, mass, sequence coverage and matched peptides from MASCOT peptide identification **(C)** representative mass spectrum of AnxA2 peptide

Figure S2

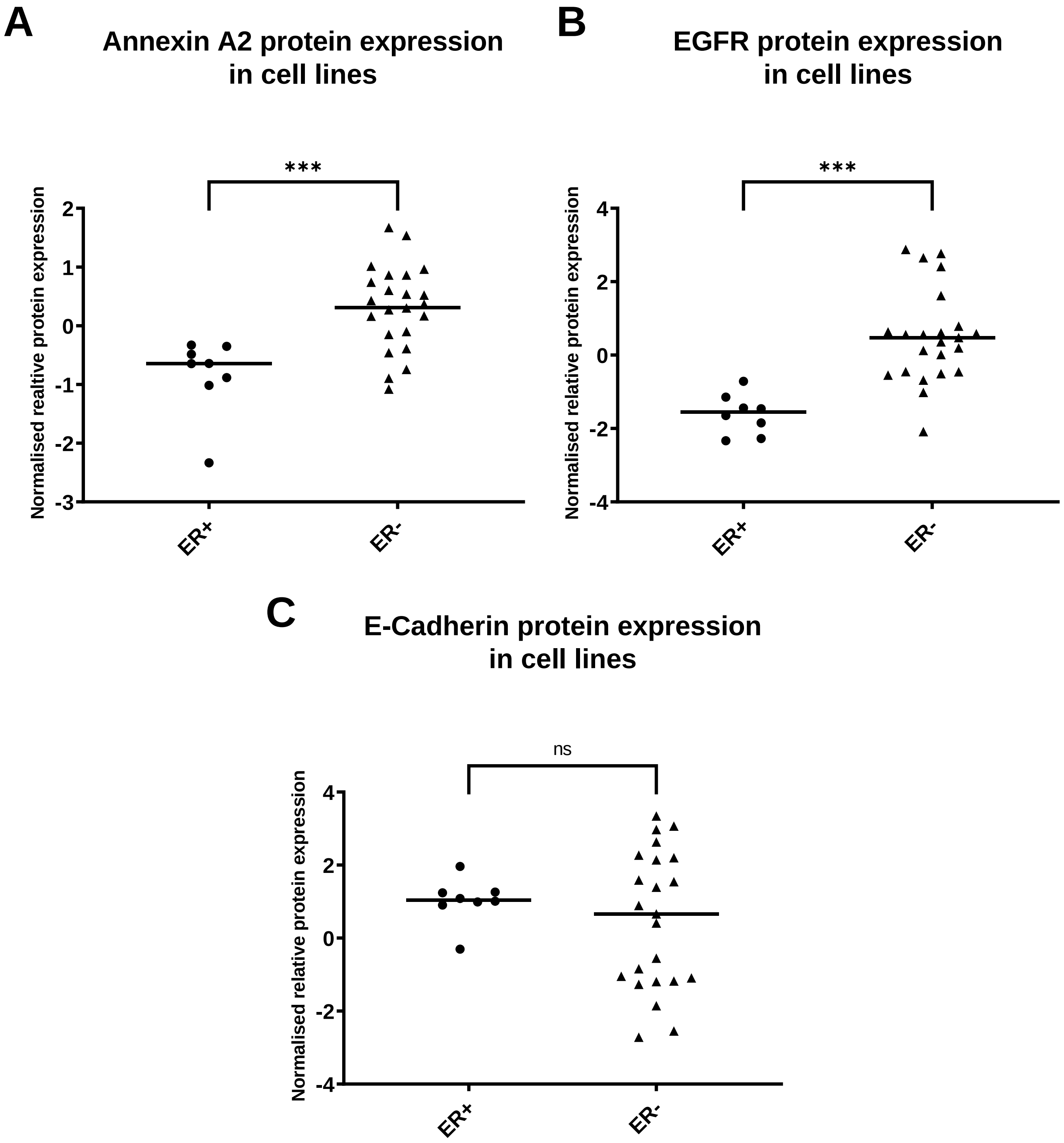

Figure S2: Graphs of Annexin A2, EGFR and E-Cadherin protein expression data from Nusinow, D. P. et al. (2020)[1], stratified according to ER status (ER+ n=8 , ER- n=23). Analysis showed (**A**) Annexin A2 (p=0.0008) and (**B**) EGFR (p <0.0001) are significantly upregulated in ER- breast cancer cell lines. **(C)** No significant difference was seen for E-Cadherin expression (p= 0.6105). Protein expression represented as normalised protein expression, as measured by quantitative proteomics. Statistical difference between groups tested using Mann Whitney U test.

[1]: Nusinow, D. P., et al. (2020). "Quantitative Proteomics of the Cancer Cell Line Encyclopedia." Cell **180**(2): 387-402 e316.

Figure S3 (A)

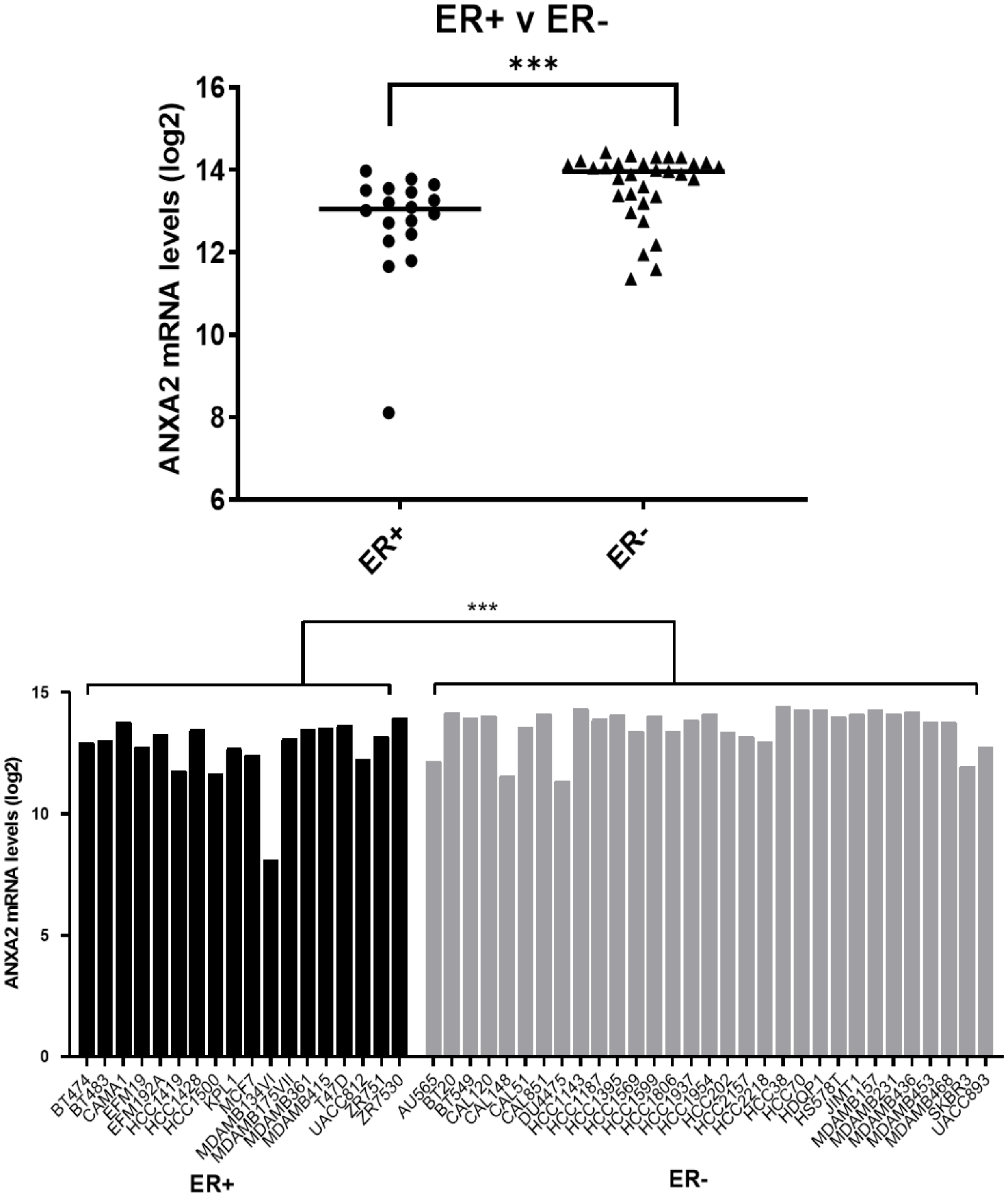

Figure S3: Publicly available Affymetrix gene expression data for human breast cancer cell lines was obtained from the CCLE [2]. Cell lines were categorised according to ER, PR and HER2 expression status and difference in ANXA2 expression between the two groups was measured using Mann-Whitney U test. (A) ANXA2 expression is higher in ER- cell lines, p = 0.0008 (B) ANXA2 expression is higher in PR- cell lines, p = 0.0244 (C) ANXA2 expression is not significantly different between HER2+ and HER2- cell lines. p= 0.0769

[2]: Barretina, J., et al. (2012). "The Cancer Cell Line Encyclopedia enables predictive modelling of anticancer drug sensitivity." Nature **483**(7391): 603-607.

Figure S3 (B)

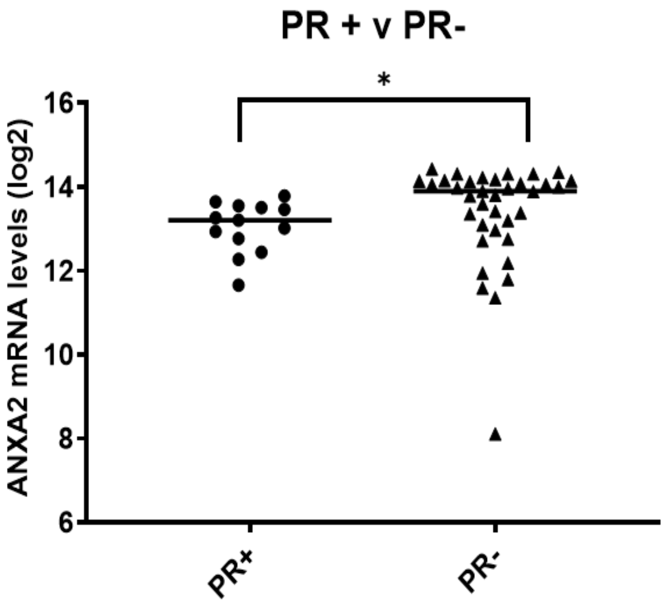

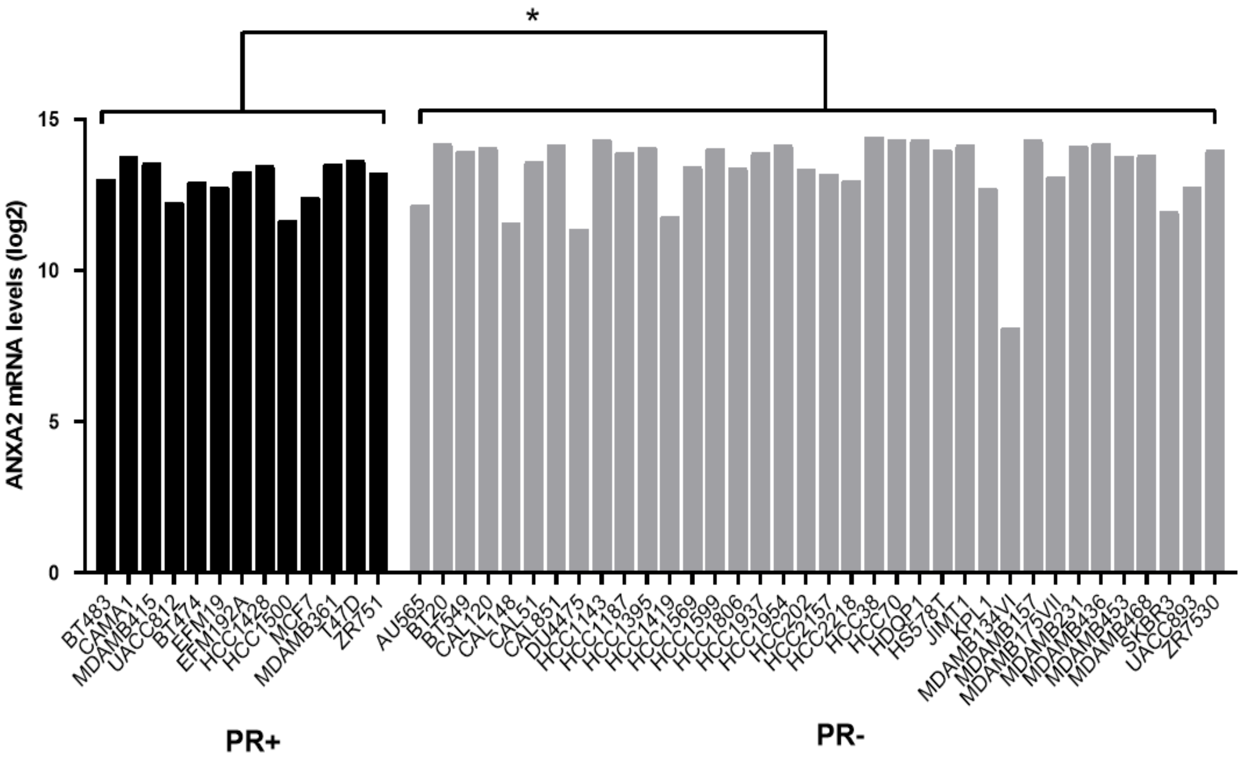

Figure S3: Publicly available Affymetrix gene expression data for human breast cancer cell lines was obtained from the CCLE [2]. Cell lines were categorised according to ER, PR and HER2 expression status and difference in ANXA2 expression between the two groups was measured using Mann-Whitney U test. (A) ANXA2 expression is higher in ER- cell lines, $p = 0.0008$ (B) ANXA2 expression is higher in PR- cell lines, $p = 0.0244$ (C) ANXA2 expression is not significantly different between HER2+ and HER2- cell lines. $p= 0.0769$

[2]: Barretina, J., et al. (2012). "The Cancer Cell Line Encyclopedia enables predictive modelling of anticancer drug sensitivity." Nature **483**(7391): 603-607.

Figure S3 (C)

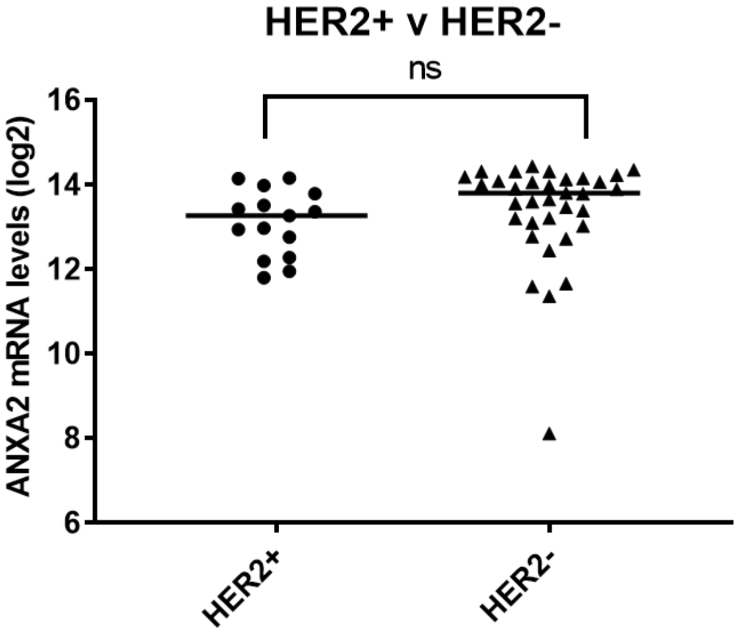

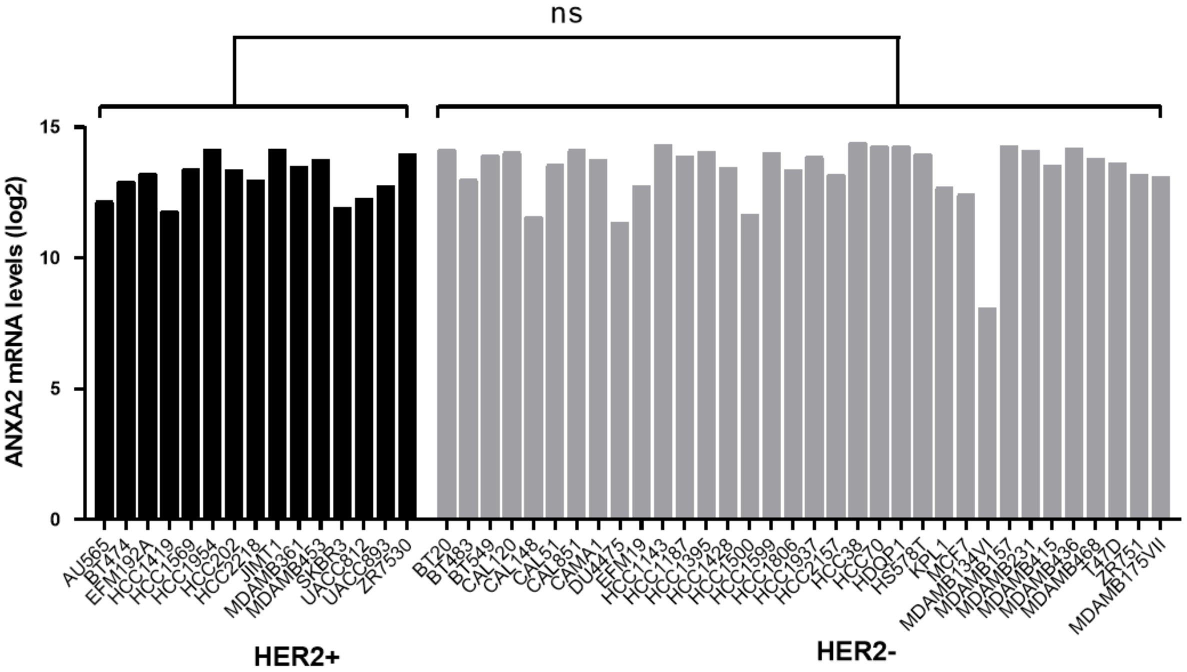

Figure S3: Publicly available Affymetrix gene expression data for human breast cancer cell lines was obtained from the CCLE [2]. Cell lines were categorised according to ER, PR and HER2 expression status and difference in ANXA2 expression between the two groups was measured using Mann-Whitney U test. (A) ANXA2 expression is higher in ER- cell lines, p = 0.0008 (B) ANXA2 expression is higher in PR- cell lines, p = 0.0244 (C) ANXA2 expression is not significantly different between HER2+ and HER2- cell lines. p= 0.0769

[2]: Barretina, J., et al. (2012). "The Cancer Cell Line Encyclopedia enables predictive modelling of anticancer drug sensitivity." Nature **483**(7391): 603-607.

Figure S4

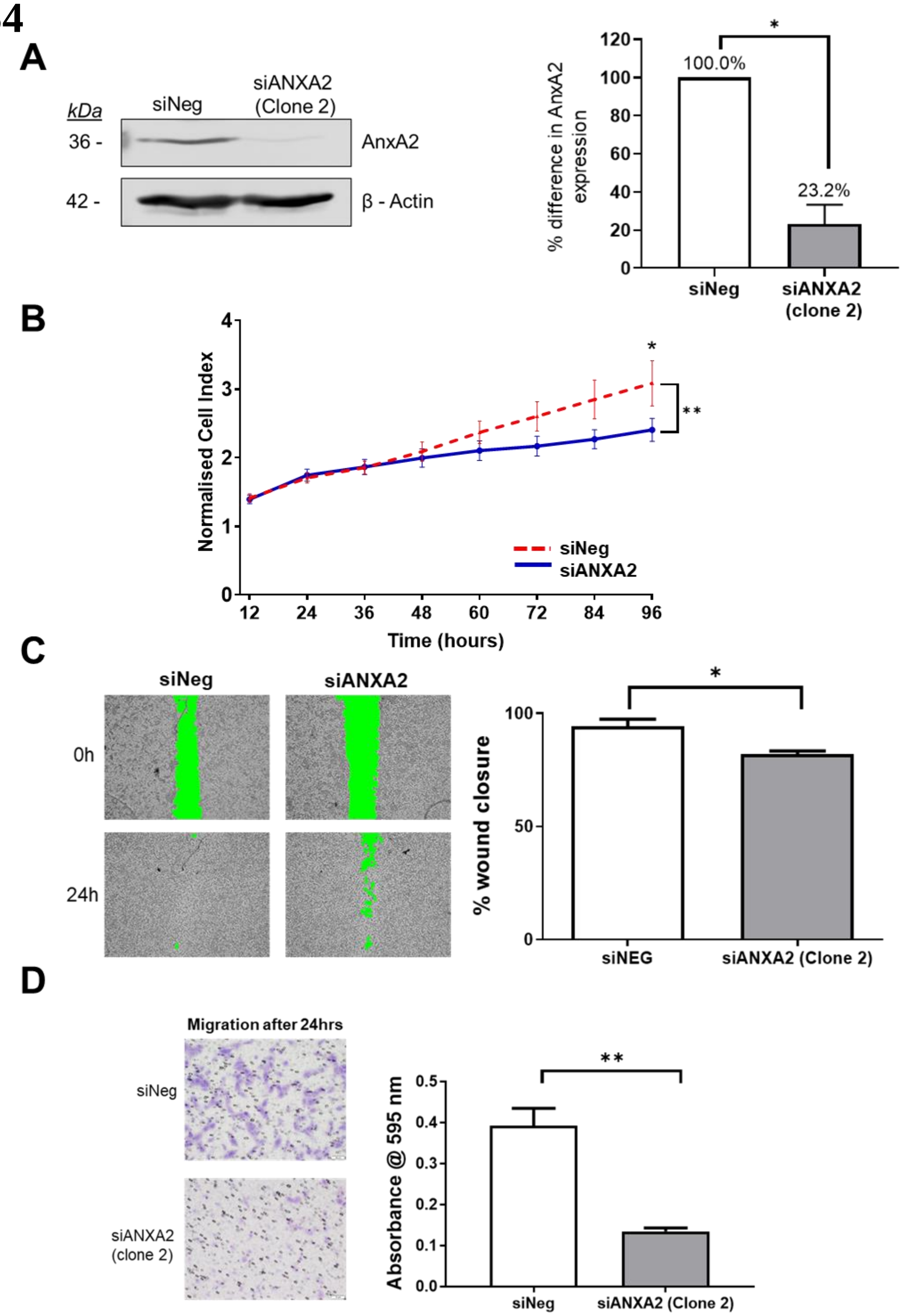

Figure S4. Additional functional assays with second siRNA clone on MDA-MB-231 cells. **(A)** Efficiency of ANXA2 knockdown (KD) with Clone 2 after 72h and densitometry analysis of Annexin A2 normalised against beta-actin loading control. **(B)** To measure cell proliferation, KD and negative control cells were seeded in duplicate into E-plate wells and the rate of proliferation was measured in real-time using the xCELLigence system. Proliferation of KD cells (blue) was compared to the proliferation of negative control wells (red) over the course of 96 hours. Data displayed as mean cell index +/- SEM, n=3. **(C)** To measure wound healing migration, knockdown and negative control cells were plated into dishes containing Ibidi Culture Inserts. After 72h, the inserts were removed to generate a cell free wound. The green zone indicates the wound size as measured at 0h and 24h post-removal of insert by imaging at 4X magnification and analysis on Image J. Data displayed as % wound closure for 3 fields of view per condition +/- SEM, p= 0.0213. (D) To measure EGF directed cell migration, cells were plated onto the upper chamber of transwell membrane and allowed to migrate towards 50ng/mL EGF chemoattractant for 24 hours. Cells were removed from the upper chamber of the membrane, leaving only those that had migrated to the lower chamber. Migrated cells were stained with crystal violet and imaged using an inverted light microscope. Crystal violet stain was dissolved and intensity measured as absorbance at 595nm in triplicate. Data displayed as mean absorbance values for 3 triplicate measurements per condition +/- SEM, p=0.0038.

Figure S5

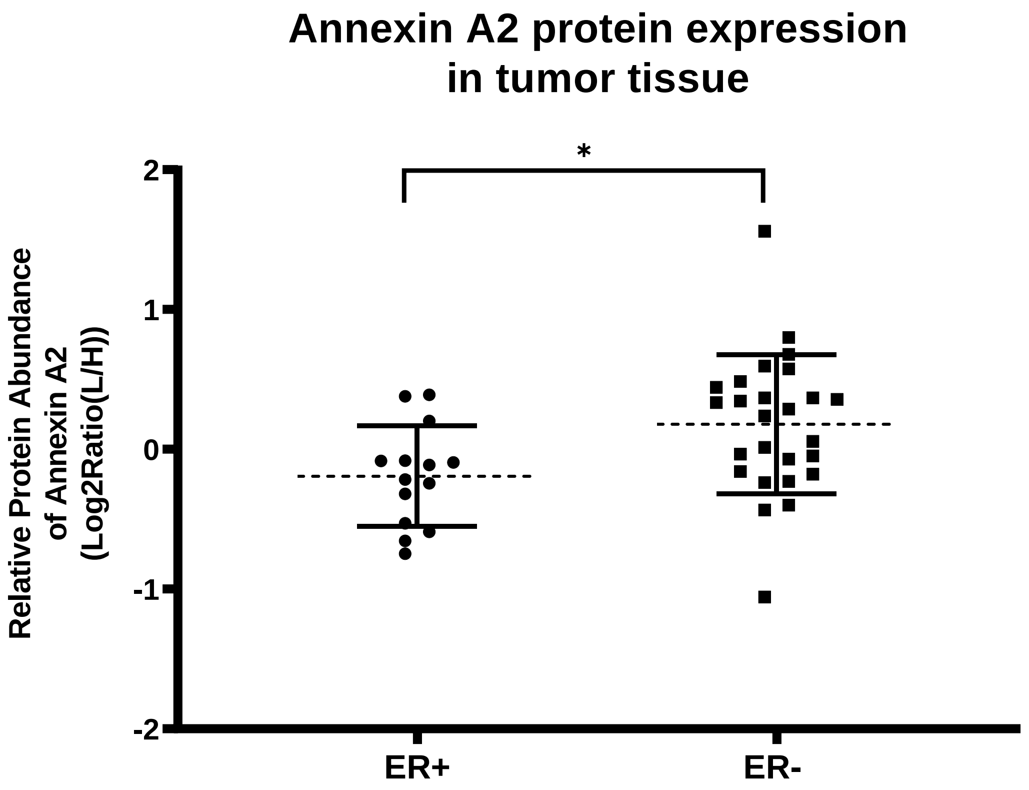

Figure S5: Graphs of Annexin A2 protein expression data in breast cancer tissue from S. Tyanova *et. al*. (2016)[3], stratified according to ER status (ER+ n=14 , ER- n=26). Analysis showed Annexin A2 is significantly upregulated in ER- breast cancer tumor tissue (p= 0.0185). Protein expression represented as log2 normalised protein expression, as measured by quantitative proteomics and SILAC labelling. Statistical difference between groups tested using Student’s t-test.

[3] Tyanova, S., et al. (2016). "Proteomic maps of breast cancer subtypes." Nat Commun **7**: 10259.

Figure S6

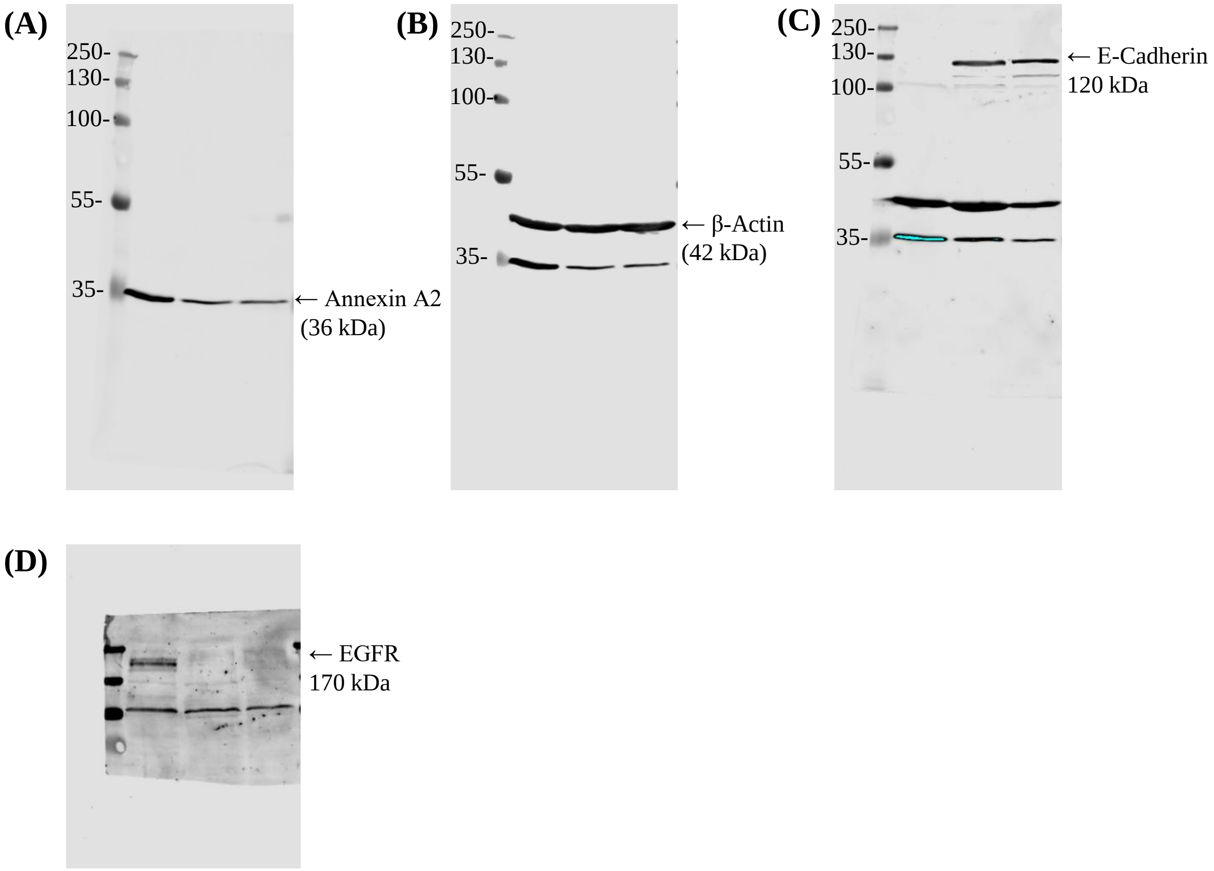

Figure S6 Full blots for Figure 3 (A) Comparison of Annexin A2 expression in three breast cancer cell lines

Displayed in order of antibody probing (**A**) Probed with Annexin A2 antibody (mouse) and IRdye700- secondary antibody **(B)** Probed with B-Actin antibody (mouse) and IRdye700- secondary antibody **(C)** Probed with E-cadherin (rabbit) and IRdye800- secondary antibody. (Overexposure on Annexin band due to repeated probings, (A) was used for densitometry) **(D)** Probed with EGFR mouse and IRdye700- secondary antibody. Blot was cut in half.

Figure S7

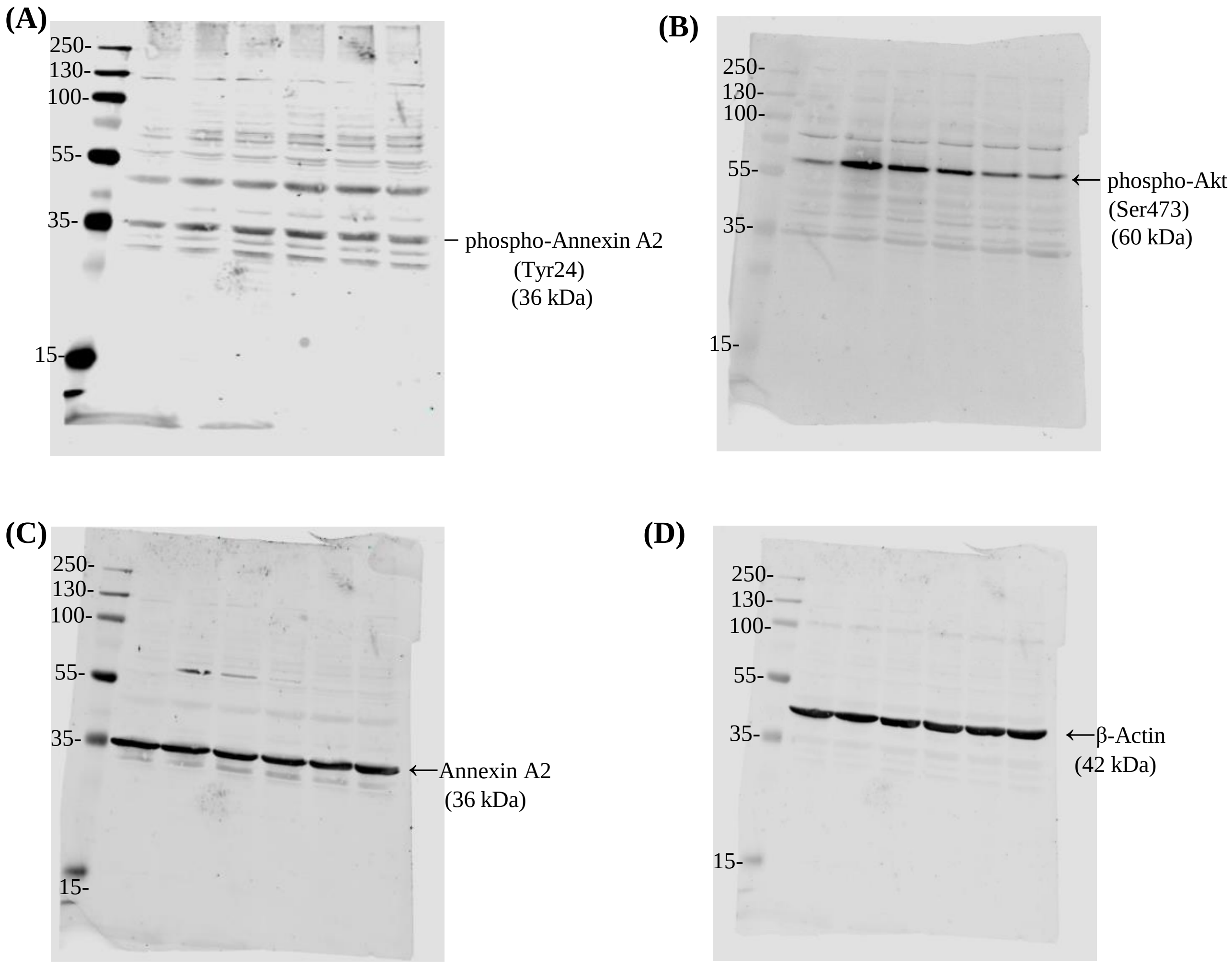

Figure S7 Full blots for Figure 3 (E) Western blot showing the effect of EGF stimulation on AnxA2 expression and phosphorylation in MDA-MB-231 cells. Displayed in order of antibody probing (**A)** Probed with phospho-Annexin A2 (Tyr24) antibody (mouse) and IRdye700- secondary antibody **(B)** Probed with phospho-Akt (Ser473) antibody (rabbit) and IRdye800- secondary antibody **(C)** Probed with Annexin A2 (Rabbit) and IRdye800- secondary antibody **(D)** Probed with B-Actin antibody (mouse) and IRdye700- secondary antibody

Figure S8

(A)

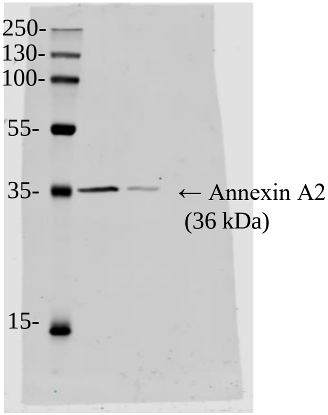

(B)

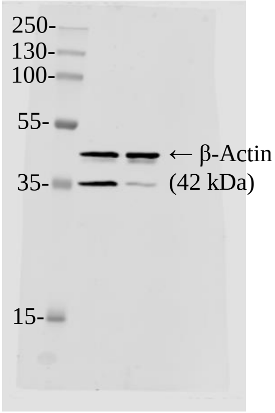

Figure S8 Full blots for Figure 4 (A) MDA-MB-231 cells were transfected with a small interference oligonucleotide against ANXA2 [10 nM] for 72 hours, using the Neon Transfection system. Representative blot showing the efficiency of ANXA2 knockdown.

Displayed in order of antibody probing (**A)** Probed with Annexin A2 antibody (mouse) and IRdye700- secondary antibody **(B)** Probed with B-Actin antibody (mouse) and IRdye700- secondary antibody

Table S1 List of newly synthesised proteins identified in 2-D migration experiment

Accession	Score	Mass	Identified protein (Gene symbol)
1568551	440	13928	HIST1H2BE
1147813	245	334023	DSP
7669492	157	36201	GAPDH
32097	153	11317	HIST2H4A
4506763	352	12276	S100A3
28336	128	42128	ACTB
3891470	186	14992	LGALS7
4505591	46	22324	PRDX1
307504	54	77121	TGM3
29789445	72	115462	DSG4
1032207174	121	14338	HIST1H2AB
4506773	64	13291	S100A9
61966777	95	44946	VSIG8
30506	49	114670	DSG1
494066	51	23438	GSTP1
35222	83	71209	HSPA6
15928913	30	21186	HSPB1
34412	30	79911	LTF
47825361	35	30942	NCCRP1
4505821	64	16847	PIP
535015	100	81637	PKP1
35959	24	50055	TUBB4A
194389794	24	61600	ANPEP
4757756	64	38808	ANXA2
619383	32	28317	APOD
38026	29	34942	AZGP1
4557367	36	53155	BLMH
13929432	16	26304	CABP1
379642577	25	19979	CALHM4
336038727	20	22696	COX1
4503143	43	45037	CTSD
14278227	47	16302	DFFA
1033647300	35	46672	GNE
20810274	16	47787	GOT1L1
533693	29	81277	GPD2
20805939	17	31353	HUS1B
455852	14	3914	ITIH2
119590706	21	54000	KIAA2012
5031857	21	36950	LDHA
179531	26	26229	LGALS3
62988340	17	61327	LRIT2
556708028	15	67365	MT-ND5
3643107	16	72739	PIAS1
2992541	19	205150	PPL
3318841	15	25011	PRDX6
34276	20	132298	PTPRC
365812818	14	4207	RBM5
29888	31	10988	S100A8
10435945	23	26222	SELENBP1
187302	19	27873	SFN
5803185	22	28889	SYPL1
440576057	18	6045	TNS4
37492	35	50810	TUBA1A
1065111	19	11629	TXN
115529037	31	6110	ZNF61

Table S2 List of newly synthesised proteins identified in 3-D invasion experiment.

Accession	Score	Mass	Identified protein (Gene symbol)
194384240	2042	59042	KRT5
4501885	385	42052	ACTB
7669492	401	36201	GAPDH
3891470	671	14992	LGALS7
158254664	217	42362	ACTC1
4506773	306	13291	S100A9
194382264	220	19161	JUP
4757756	169	38808	ANXA2
190668	114	11564	S100A7
1025735746	59	49249	TUBA1A
4885111	164	16937	CALML3
7673316	132	15911	CALML5
338695	35	50240	TUBB
14278227	136	16302	DFFA
307504	99	77121	TGM3
1004171516	52	12003	HIST1H3A
186837	48	205178	LAMB1
32015	45	50503	TUBA4A
1552516	38	27092	PRSS3P2
5174661	35	11206	S100A2
29446	32	16070	HBB
5031857	29	36950	LDHA
38026	57	34942	AZGP1
4557581	50	15497	FABP5
494066	37	23438	GSTP1
7329718	36	257758	VPS13D
37492	34	50810	TUBA1A
47825361	33	30942	NCCRP1
4885165	30	11000	CSTA
37183160	29	19588	ZG16B
4504165	27	86043	GSN
296734	27	19590	PSMB6
37403	26	73052	NTRK1
3170180	26	49156	SDCCAG8
5457357	25	88884	AFG3L2
35222	25	71209	HSPA6
18098558	24	99380	PIWIL1
164598948	23	32098	ABO
657232307	22	12915	PPL
22760940	21	22072	PERP
21755948	20	55672	A2ML1
215261502	20	25318	SPTAN1
307265	19	30421	HLA-DRB1
4261629	19	5820	PIGA
13540600	18	38523	NDEL1
5803225	17	29326	YWHAE
1125020	17	58289	ZRSR2P1
34226	16	297083	LAMA1
16549271	16	41471	PPM1K
75750526	15	18654	DUSP28
1203796	15	56567	STK3
15928896	15	76589	SYTL4
3360457	14	39478	CUL3
54696744	14	30831	DCK
4507503	14	56244	KLF11
6912680	14	44964	SPO11

Table S3. Sequences of the small interfering RNA used for ANXA2 knockdown.

Clone no.	Target sequence	Product no.	Working Concentration
1	CGGCAAGTCCCTGTACTATTA	Hs_ANXA2_8	10nM
2	CACGGCCTGAGCGTCCAGAAA	Hs_ANXA2_10	10nM

Table S4: Clinical information of local breast cancer cohort (n=30)

Mean age (±sd)	57.5 (±12.1)
Unknown	5
Histology	
Ductal	22
Lobular	2
Mixed	2
Other	1
Unknown	3
Stage	
I	4
II	15
III	7
IV	0
O	1
Unknown	3
Grade	
1	1
2	18
3	7
Unknown	4
Lymph node	
Positive	14
Negative	13
Unknown	3
ER status	
Positive	24
Negative	2
Unknown	4
PR status	
Positive	16
Negative	8
Unknown	6
HER-2 status	
Positive	1
Negative	24
Unknown	5

Table S5: Clinical information of GSE42568 breast cancer cohort (n=104)

Mean age (±sd)	58.8 (±11.8)
Grade	
1	11
2	40
3	53
Lymph node	
Positive	59
Negative	45
ER status	
Positive	67
Negative	34
Unknown	3
